# Supplementary material for: MyPal-Child study protocol: an observational prospective clinical feasibility study of the MyPal ePRO-based early palliative care digital system in paediatric oncology patients
Source: BMJ Open. 2021 Apr 13;11(4):e045226. doi: 10.1136/bmjopen-2020-045226 (PMC8051393; doi:10.1136/bmjopen-2020-045226)
Supplement: Supplementary data [file bmjopen-2020-045226supp001.pdf]

Version 1.1 – 05.03.2020

Informed Consent Form for Parents / Legal Guardian

- I consent to use Internet connected devices, e.g. smart phone or tablet, to complete self-reporting questionnaires monthly via an app to assess well-being, satisfaction with cancer care received for my child and their impact on the family.
- I consent to respond one-time to a paper-based questionnaire 7 days after the 6 months of study enrollment to evaluate the usability of the MyPal platform.
- I consent that I have read and understand the information sheet explaining the above research project and I have had the opportunity to ask questions about it.
- I consent that information has been provided to me in a language that I fully understand.
- I am aware that NO biological samples will be collected from my child in the study and that the study does NOT entail the administration of any medication to my child.
- I am aware that the participation of my child is absolutely voluntary and that I am free to withdraw my consent at any time without giving any reason and without there being any negative consequences.
- I consent that if I withdraw myself or my child from the study after some data have been collected, these data will be anonymized but not deleted. No further data will be collected in this case.
- I am aware that in all research outputs (papers, presentations, articles, reports) the anonymity the data will be protected.
- I am aware that data collected during MyPal project will never be transferred to countries outside of the European Union.
- I consent that medical data that has been obtained during this project will be stored for 15 years, in (name of the clinical site) and the data that has been collected for the purposes of the study, will be kept in a de-identified form at the central installation of the sponsor of the study.
- I consent that health care professionals will report disease-related data of your child to the MyPal system.
- I am aware that any supportive information provided by health care professionals (e.g. physicians) via the MyPal platform does not imply legal liability and that MyPal is not designed as an emergency alert system and doctors might not respond immediately.
- I consent that I have understood the information sheet for the study including information on rights for affected person in terms of personal data. Hereby, I have obtained the contact details of the Data Protection Officer responsible, in case I have concerns or queries explicitly dealing with data protection or want to lodge a complaint due to this issue.

I have read and understood this consent form and I have been provided with information regarding the research study. I have been given a copy of the information sheet and of this consent form.

In case I have concerns of any kind or further questions, I may contact (enter contact details)

A copy of this agreement will be given to me and another copy will be retained for record keeping by the project.

○ **I agree for my child to participate in the study**

Name of the child: ____________________________________, Birthday: ___.___.20….

______________________	______________	______________________
Name of the Mother	Date	Signature
______________________	______________	______________________
Name of the Father	Date	Signature
______________________	______________	______________________
Name of the Researcher	Date	Signature

Meyerheim M, *et al. BMJ Open* 2021; 11:e045226. doi: 10.1136/bmjopen-2020-045226
