# Supplementary material for: MyPal-Child study protocol: an observational prospective clinical feasibility study of the MyPal ePRO-based early palliative care digital system in paediatric oncology patients
Source: BMJ Open. 2021 Apr 13;11(4):e045226. doi: 10.1136/bmjopen-2020-045226 (PMC8051393; doi:10.1136/bmjopen-2020-045226)
Supplement: Supplementary data [file bmjopen-2020-045226supp002.pdf]

Version 1.1 – 05.03.2020

MyPal-Child Information Sheet for Parents / Legal Guardian

We would like to invite your child to participate in a study conducted by Dr (enter authorized clinician) and his/her team at the University of (enter Clinical Site). Before you decide to give permission for your child, we would like to help you understand the study and what is involved. Please take time to read the following information carefully and to decide whether or not you wish your child to take part.

In this study we are interested in exploring the use of digital technology that will help you (and possibly members of your family) to communicate your condition more accurately and effectively to your health care providers (i.e. oncologists, specialized physicians, psychologists, nurses). The aim is to improve the quality of care by using modern methods of individualized information, communication and support for patients with cancer the main focus of which is the patient (Patient-Reported Outcome). Supportive information provided by health care professionals via the application do not imply legal liability.

Aims of the study: The study in the context of the EU project MyPal[1] aims to propose a new system of care that is anticipated to improve quality of life in children with cancer and their parents, by exploring how digital technology can enhance communication, improve decision-making, emotional support and reinforcement of young patients and reduce symptom burden. It only involves observation of your child regarding the use of an application in a gamified form. It is important for you to know that the study:

- does not involve collection of biological samples
- does not involve administration of any medication
- does not aim to provide or change medical treatment solely based on the MyPal platform
- MyPal is not an emergency alert system and doctors may not respond immediately

Why has your child been chosen: Your child has been chosen to participate in this study because he /she is between 6-17 years of age and has been diagnosed with acute leukemia or solid cancer for which anti-cancer treatment is already being provided. Also, because he/she is fit to participate in the study and able to use an Internet connected device (smartphone or tablet). The contribution of children and adolescents is very important for the study to explore their specific needs and preferences and with the aim of making young people with cancer stronger to deal with their condition.

What does your child have to do: If you and your child agree to participate, your child will receive an introduction to get familiar with the MyPal App and its tools and how to use them. For the upcoming 6 months, your child can then perform the following tasks:

- Use the MyPal app through Internet connected devices, such as smart phone, tablet for a game which involves questionnaires about their well-being and symptoms. In addition, the physical activity of your child will be roughly estimated based on a daily step counter.
- With regard to the various questionnaires involved, the child will be free to choose not to answer any particular question or questions. Completion will be scheduled at baseline, and then monthly, some of them several times a week, until the end of the study. It is possible that these time intervals may change due to the needs of the study.
- Respond only once to paper-based questionnaire 7 days following the 6 months of study enrollment in order to evaluate the usability of the MyPal platform.

During the completion of the questionnaires your child may get assistance by you, a member of our research team or a health care professional.

Which tasks you as parents/legal guardian will be asked to complete:

- You will also receive an introduction on how to get familiar with and use the MyPal platform and its tools.
- Use the MyPal platform to complete monthly questionnaires about your well-being, satisfaction with your child's received care and the impact of the disease on you and your family.
- Help your child completing questionnaires if they ask you for help.
- respond only once to a paper-based questionnaire 7 days following the 6 months of study enrollment in order to evaluate the usability of the MyPal platform.

[1] Available from: https://mypal-project.eu/. Last accessed: January 20, 2019

Will the data collected in this project be kept confidential? Data protection is one of our most important priorities in this study. National laws on personal data protection will be implemented in order to guarantee the highest standards in personal data management. Further, all procedures for protecting personal information in this study are in accordance with the approved rules of the University of (enter clinical site) and with the European legislation including the General Data Protection Regulation 2016/679 (GDPR). Only data that is necessary for this research and no additional data will be collected. Our technology partners will provide technical support and tools such that data protection and security requirements are ensured. Any information, that is obtained in this study and that can be assigned to you or your child will remain confidential.

If you agree for your child to take part in this study, we will use his/her data in the ways needed to conduct our study and analyse the study results. Your rights to access, change or move your child's information are limited, as we need to manage this information in specific ways in order for the research to be reliable and accurate (cf. point d of Article 17(3) GDPR and Article 89 GDPR). If your child withdraws from the study, we will keep the information that we have already obtained and anonymize it for analysis at the end of the study. Anonymization means that the stored data is altered in such a way that it cannot be restored in its original form and all information which could link the collected data to you or your child is deleted. No more data will be collected after withdrawal.

We will not allow people from outside the project to know how you or your child answered the questions within the study. In case you have concerns or queries explicitly concerning data protection or you want to get into contact or lodge a complaint with data protection authorities, you can also contact the Data Protection Officer of our Research Institution: the corresponding contact details can be found on the last page.

Storage of data and security: Data will be stored in two ways, locally and centrally, which is elucidated in the following.

The app installed on the mobile device stores encrypted data, i.e. answers to questionnaires, only temporarily locally if the mobile device has no internet connection. As soon as an internet connection is available, the answers will be transmitted to the local database at your hospital and deleted from the mobile device. After the initial login to the app, an access token is stored on the device which facilitates each upcoming login.

The local database of (name of the clinical site) will store:

- responses to questionnaires about symptoms, spontaneous symptom reporting forms, etc.
- personal data (patient name, provenance, treating clinical site)
- clinical information (gender, age, diagnosis, disease stage/risk, treatment scheme, expected outcome, functional impairment, etc.). Concerning the clinical information, the (a) age and (b) diagnosis of the study subject are categorized before storage instead of precise detail.

The data included in the local database will be accessible only by the team of local investigators, and even the members of the team will only have access to the information needed for the specific user category with regard to the study. All personally identifiable data remain at any time at the (enter clinical site) and will neither be shared with the other participating clinical sites nor study sites. Furthermore, the data is protected against access by unauthorized people by username and password.

The central database which resides at the side of the sponsor of the study (Centre for Research and & Technology Hellas, Greece) and which fulfills all technical and organizational requirements for the safety and the security of the stored data to protect them against unauthorized people's access by access restriction. The central database will include a part of the local data (responses to the questionnaires and information needed to assess the endpoints of the study, e.g. quality of life, satisfaction with care etc.) which will be transferred from the participating clinical center to the central database identification to enable the joint analysis of data from the participating clinical sites. Before the transmission, personal data is removed (Anonymization). Anonymization is performed by removing the patient's name from the personal data and keeping among the clinical data only the gender, age group and categorized diagnosis information.

All data obtained in the study will be maintained for 15 years in the (enter name of the clinical site) where they have been collected or created.

Does your child have to participate? Participation is completely voluntary and you are free to consent or not to the participation of your child. Even if you agree, you remain free to withdraw this consent at any time without giving any reason and without this affecting your or the child's relationship with our research team or with the team providing clinical care. If you decide to withdraw from the study once data collection commenced, the collected data will not be erased, but will be retained in the study but in an anonymized form which will not in any way permit the child's or your identification. Once the research has been completed and the data been analysed, it will not be possible to withdraw this data from the study. No more data will be collected from you or your child after the withdrawal.

Are there possible disadvantages and/or risks in taking part? Participation of your child does not entail any risk of discomfort, pain, injury, illness or disease and we remind you that the study itself does NOT involve collection of biological samples or administration of any medication. However, if we come across an unexpected finding which we think requires notifying you, we plan to inform you, to discuss this in our team, and to consult with your child's treating physician. If you have any concerns please feel free to contact the Principal Investigator (see below for details) and once again, we would like to remind you that your participation is entirely voluntary. The health care professionals do not bear liability for information which are provided to the patients or parents via the Apps.

What are the possible benefits of taking part? You, your child, or your family may not receive any personal benefits from participating in the study except from the possibility to use and play the game. However, you may find participation in the study is a positive experience, through the use of the apps and by contributing to the improvement of the quality of life of young cancer patients in the process of their treatment.

Transfer of data: All data collected during the MyPal project will not be transferred to countries outside of the European Union.

What will happen to the results of the research project? The results will be used only for research purposes; they may be reported in research publications and may be made available to other researchers in an anonymized form, e.g. in presentations. In every research output (papers, presentations, articles, reports) the complete anonymity of your data will be ensured.

Right of the individual affected in terms of processing personal data

Based on the EU Data Protection Basic Regulation 2016/679 (GDPR), you are entitled the following data protection rights for individuals affected, which you can assert against the involved hospital operators.
You have the right of access to stored personal data relating to you (Article 15 GDPR). If you discover that incorrect data concerning your person is being processed, you can request correction or purpose-related supplementation (Article 16 GDPR).
You have the right to request the deletion of your data if there are specific reasons for deletion. This is particularly the case if they are no longer necessary for the purpose for which they were originally collected or processed (Article 17 GDPR).
You have the right to restrict the processing of your data, which means that your data will not be deleted but will be for data portability (Article 20 GDPR).
In principle, you also have a general right of objection to lawful data processing which is of public interest, in the exercise of official authority or on the basis of the legitimate interest of a site (Article 21 GDPR).
As already mentioned, however, these rights may be restricted in accordance with point d of Article 17(3) GDPR and Article 89 GDPR if they make it impossible or seriously impair the achievement of the study objectives.

Complaint to the supervisory authority in case of data protection violations

You have the right to forward a complain to the supervisory authority if you think that your personal data is being processed unlawfully. The address of the supervisory authority responsible for (specify clinical partner) is:

The State Commissioner for Data Protection (give details of the respective State Commissioner for Data Protection + insert address)

Contact with the data protection officer of (specify clinical partners) is as follows:

(please specify address, e-mail, telephone)

The contact to the local study director project at (indicate clinical partner) is as follows:
(indicate corresponding address/email/telephone number WITHOUT mobile phone number)

If you have any questions or concerns regarding the study, please do not hesitate to contact him/her at any time for further information.

Ethical approval: This study has obtained a positive vote by the Ethics Committee of the University of (enter clinical site) which means that the conduction of the study does not give rise to any objections from the ethical point of view.
