# Supplementary material for: MyPal-Child study protocol: an observational prospective clinical feasibility study of the MyPal ePRO-based early palliative care digital system in paediatric oncology patients
Source: BMJ Open. 2021 Apr 13;11(4):e045226. doi: 10.1136/bmjopen-2020-045226 (PMC8051393; doi:10.1136/bmjopen-2020-045226)
Supplement: Author's manuscript [file bmjopen-2020-045226.draft_revisions.pdf]

BMJ Open

BMJ Open is committed to open peer review. As part of this commitment we make the peer review history of every article we publish publicly available.

When an article is published we post the peer reviewers' comments and the authors' responses online. We also post the versions of the paper that were used during peer review. These are the versions that the peer review comments apply to.

The versions of the paper that follow are the versions that were submitted during the peer review process. They are not the versions of record or the final published versions. They should not be cited or distributed as the published version of this manuscript.

BMJ Open is an open access journal and the full, final, typeset and author-corrected version of record of the manuscript is available on our site with no access controls, subscription charges or pay-per-view fees (http://bmjopen.bmj.com).

If you have any questions on BMJ Open's open peer review process please email info.bmjopen@bmj.com

BMJ Open

The MyPal-Child study protocol: an observational prospective clinical feasibility study of the MyPal ePRO-based early palliative care digital system in paediatric oncology patients

Journal:	*BMJ Open*
Manuscript ID	bmjopen-2020-045226
Article Type:	Protocol
Date Submitted by the Author:	25-Sep-2020

Complete List of Authors:	Meyerheim, Marcel; Saarland University Hospital and Saarland University Faculty of Medicine, Clinic of Pediatric Oncology and Hematology<br>Karamanidou, Christina; Centre for Research and Technology-Hellas, Institute of Applied Biosciences<br>Payne, Sheila; Lancaster University, International Observatory on End of Life Care, Institute for Health Research<br>Garani-Papadatos, Tina; University of West Attica, Department of Public Health Policy<br>Sander, Annette; Hannover Medical School, Clinic for Paediatric Oncology and Haematology<br>Downing, Julia; International Children's Palliative Care Network<br>Stamatopoulos, Kostas; Centre for Research and Technology-Hellas, Institute of Applied Biosciences<br>Ling, J.; European Association for Palliative Care, Head Office<br>Payne, Cathy; European Association for Palliative Care, Head Office<br>Scarfò, Lydia; Università Vita Salute San Raffaele, Department of Onco-Haematology, Division of Experimental Oncology<br>Lokaj, Petr; University Hospital Brno, Department of Pediatric Oncology<br>Maramis, Christos; Centre for Research and Technology-Hellas, Institute of Applied Biosciences<br>Graf, Norbert; Saarland University Hospital and Saarland University Faculty of Medicine, Clinic of Pediatric Oncology and Hematology

Keywords:	Paediatric oncology < PAEDIATRICS, Paediatric palliative care < PALLIATIVE CARE, Health informatics < BIOTECHNOLOGY & BIOINFORMATICS

SCHOLARONE™
Manuscripts

I, the Submitting Author has the right to grant and does grant on behalf of all authors of the Work (as defined in the below author licence), an exclusive licence and/or a non-exclusive licence for contributions from authors who are: i) UK Crown employees; ii) where BMJ has agreed a CC-BY licence shall apply, and/or iii) in accordance with the terms applicable for US Federal Government officers or employees acting as part of their official duties; on a worldwide, perpetual, irrevocable, royalty-free basis to BMJ Publishing Group Ltd ("BMJ") its licensees and where the relevant Journal is co-owned by BMJ to the co-owners of the Journal, to publish the Work in this journal and any other BMJ products and to exploit all rights, as set out in our licence.

The Submitting Author accepts and understands that any supply made under these terms is made by BMJ to the Submitting Author unless you are acting as an employee on behalf of your employer or a postgraduate student of an affiliated institution which is paying any applicable article publishing charge ("APC") for Open Access articles. Where the Submitting Author wishes to make the Work available on an Open Access basis (and intends to pay the relevant APC), the terms of reuse of such Open Access shall be governed by a Creative Commons licence – details of these licences and which Creative Commons licence will apply to this Work are set out in our licence referred to above.

Other than as permitted in any relevant BMJ Author's Self Archiving Policies, I confirm this Work has not been accepted for publication elsewhere, is not being considered for publication elsewhere and does not duplicate material already published. I confirm all authors consent to publication of this Work and authorise the granting of this licence.

For peer review only

The MyPal-Child study protocol: an observational prospective clinical feasibility study of the MyPal ePRO-based early palliative care digital system in paediatric oncology patients

Marcel Meyerheim[1], Christina Karamanidou[1], Sheila Payne, Tina Garani-Papadatos, Annette Sander, Julia Downing, Kostas Stamatopoulos, Julie Ling, Cathy Payne, Lydia Scarfò, Petr Lokaj, Christos Maramis and Norbert Graf[2]

[1] contributed equally to this paper, [2] corresponding author

Co-first authors

Marcel Meyerheim
Clinic for Paediatric Oncology and Haematology
Saarland University Hospital and Saarland University Faculty of Medicine
Homburg, Germany
marcel.meyerheim@uks.eu

Christina Karamanidou
Institute of Applied Biosciences
Centre for Research and Technology Hellas
Thermi Tessaloniki, Greece
ckaramanidou@certh.gr

Contributing Authors

Sheila Payne
International Observatory on End of Life Care
Lancaster University
Lancaster, United Kingdom
s.a.payne@lancaster.ac.uk

Tina Garani-Papadatos
Department of Public Health Policy
University of West Attica
Athens, Greece
sgarani@uniwa.gr

Annette Sander
Clinic for Paediatric Oncology and Haematology
Hannover Medical School
Hannover, Germany
sander.annette@mh-hannover.de

Julia Downing
International Children's Palliative Care Network
Bristol, United Kingdom
julia.downing@icpcn.org

For peer review only

Kostas Stamatopoulos
Institute of Applied Biosciences
Centre for Research and Technology Hellas
Thermi Tessaloniki, Greece
kostas.stamatopoulos@certh.gr

Julie Ling
Head Office
European Association for Palliative Care
Vilvoorde, Belgium
julie.ling@eapcnet.eu

Cathy Payne
Head Office
European Association for Palliative Care
Vilvoorde, Belgium
cathy.payne@eapcnet.eu

Lydia Scarfò
Department of Onco-Haematology, Division of Experimental Oncology
Universitá Vita Salute San Raffaele
Milan, Italy
scarfo.lydia@hsr.it

Petr Lokaj
Department of Pediatric Oncology
University Hospital Bnro
Brno, Czech Republic
lokaj.petr@fnbrno.cz

Senior Author

Christos Maramis
Institute of Applied Biosciences
Centre for Research and Technology Hellas
Thermi Thessaloniki, GREECE
E-Mail: Christos.MARAMIS@ec.europa.eu

Corresponding Author and Senior Author

Norbert Graf
Clinic for Paediatric Oncology and Haematology
Saarland University Hospital and Saarland University Faculty of Medicine
Kirrbergerstraße 9
66424 Homburg, GERMANY
E-Mail: norbert.graf@uks.eu

For peer review only

Word Count

3550 words excluding title page, abstract, references, figures and table

Keywords

- Patient-Reported Outcome (PRO)
- electronic Patient-Reported Outcome (ePRO)
- Patient-Reported Outcome Measure (PROM)
- Palliative Care for Children
- Supportive Care for Children
- Serious Game
- Mobile Applications
- Observative Study

ABSTRACT

Introduction

Electronic patient-reported outcomes (ePROs) have tremendous potential to optimise palliative and supportive care for children with cancer, their families and healthcare providers. Particularly these children and their families are subjected to multiple strains caused by the disease and its treatment. The MyPal digital health platform is designed to address these complex demands by offering pursuant ePRO-based functionalities via two mobile applications, one developed for children and the other for their parents.

Methods and Analysis

In this observational prospective feasibility study, 100 paediatric oncology patients aged between 6 and 17 years and at least one of their parents/legal guardians will be recruited at three clinical sites in two European countries (Germany and Czech Republic). They will use the mobile applications which are part of the novel digital health platform. During a 6-month study period, participants will complete various ePROs via the applications addressing quality of life, satisfaction with care and impact of the disease on the family at monthly intervals. Additionally, priority-based symptom reporting is integrated into a serious game for children. Outcomes that will be assessed concern the feasibility and the evaluation of the newly designed digital health platform to contribute to the evidence base of clinical ePRO use in paediatric oncology and palliative care process.

Ethics and Dissemination

The integration of ePROs dealing with health issues into mobile applications raises ethical aspects which have been a crucial pillar in the implementation plan of this project. Particular attention has been paid regarding the design of the MyPal-Child study protocol which has obtained ethical approval from the competent ethics committees at all participating clinical sites. The dissemination and

For peer review only

exploitation of the study throughout the duration of the project as well as data processing will take place according to the guidelines agreed upon by the project's consortium in the study protocol.

Article Summary

Trial Registration Number

Universal Trial Number (UTN): U1111-1251-0043

German Clinical Trials Register (DRKS): DRKS00021458

ClinicalTrials.gov: NCT04381221

Strengths and Limitations of this study

- Multicentre (3 clinical sites in 2 European countries) observational study on the feasibility and acceptability of using ePRO-based systems in palliative care for paediatric oncology patients.
- Patient outcomes are reported independently in an inpatient/outpatient setting and the impact of this approach will be evaluated by the treating healthcare professionals.
- The design of the study is the product of a joint effort of interdisciplinary expertise from clinicians, palliative care experts, software engineers and scientists.
- Future randomised controlled trials are required to investigate the effectiveness of ePROs in paediatric palliative care management and treatment.

1. Introduction

According to the WHO definition, paediatric palliative care for children and their families is defined as "the active total care of the child's body, mind and spirit, and also involves giving support to the family. It begins when illness is diagnosed, and continues regardless of whether or not a child receives treatment directed at the disease. Health providers must evaluate and alleviate a child's physical, psychological, and social distress. Effective palliative care requires a broad multidisciplinary approach that includes the family and makes use of available community resources; it can be successfully implemented even if resources are limited. It can be provided in tertiary care facilities, in community health centres and even in children's homes" [1]. Unsurprisingly, this definition applies to children with cancer who require complex multimodal anticancer treatments but also benefit from palliative care supporting both the patients and their families to manage symptoms of the disease, side effects of treatment as well as treatment-related toxicity. Palliative care is not limited to end-of-life treatment

For peer review only

but preferably starts close to the time of diagnosis and continues during as well as outside the acute disease treatment phase to improve the quality of life (QoL). Such an early integration of palliative care either along with anticancer treatment or even as the only means of treatment in rare cases, can improve both patient and caregiver outcomes [2]. The latter is particularly relevant for parents and siblings of children or adolescents with cancer who are also impacted by the disease, its treatment as well as its prognostic uncertainty.

As children with cancer and their carers often believe that the adverse effects of anticancer treatment are inevitable, they often downplay symptom severity during clinical interviews [3], it is therefore possible, that more accurate assessments can optimise symptom reporting and generate richer and more reliable data. In that respect, major improvements can be anticipated through standardising patient-reported outcomes (PROs), defined as "measurements based on a report that comes directly from the patient about the status of a patient's health condition without amendment or interpretation of the patient's response by a physician or anyone else" [4, 5].

Overall, the use of PROs has become a prominent topic in healthcare innovation, highlighting the role of the patients' experience of their status as a key measure of healthcare quality, especially in cancer [6]. PROs provide a more comprehensive view on the patient's experience outside the environment of care and complement data gathered by HCPs or physicians. On these grounds, solutions are evidently required for facilitating the use of PROs in routine clinical practice of paediatric oncology and palliative care [7, 8], and these can be offered, amongst others, by information and communication technologies (ICT). Published evidence also supports the incorporation of electronic PRO (ePRO) assessments into healthcare as these have the potential to improve the quality of care delivered to patients with cancer [9].

MyPal is a Horizon 2020 Research & Innovation Action aiming to foster palliative care for people with cancer by leveraging ePRO systems through their adaptation to the personal needs of the person with cancer and his/her caregiver(s). It aspires to empower people with cancer and their carers in capturing more accurately their symptoms/conditions, communicate them in a seamless and effective way to their healthcare providers and, ultimately, foster action through advanced methods of identification of important deviations relevant to the patient's state and QoL. Providing this information in a timely and comprehensive manner throughout the disease course will reinforce the potential of applying a patient-centred and integrated palliative care approach for cancer patients with the participation of all relevant healthcare professionals (i.e. oncologists, specialised physicians, psychologists, nurses) to cope with the specific disease.

In order to accomplish its mission, MyPal will exploit technological advances in ICT for supporting patients, family members and healthcare providers through a systematic and comprehensive ePRO-

For peer review only

based digital health platform. It will demonstrate and validate the proposed add-ons to care for two different patient groups, i.e. adults suffering from haematological malignancies and children with solid tumours or leukaemia, hence targeting different age groups and cancer types, through two carefully designed clinical studies that will be conducted in diverse healthcare settings across Europe. The observational study with children with cancer (namely MyPal4Kids) is the subject of the presented protocol.

2. Methods and Analysis

Study Design

This is a multi-national non-experimental observational prospective feasibility study, enrolling paediatric oncology patients as well as at least one of their parents or legal guardians at 3 clinical sites in Europe. The emphasis is on data collection from the study participants via the functionalities of the newly developed MyPal digital health platform. The methodological approach involves two main mobile applications (see Section MyPal Digital Health Platform). The first comprises of a so-called 'serious game' developed with the aim to combine the advantages of modern ePROs with motivational gaming by wrapping the symptom assessment process in an entertaining cover of an age-appropriate, non-violent game. The second concerns convenient self- and proxy reporting for carers, i.e. in the context of this study the child's parents/legal guardians and healthcare providers, via ePROs as outcome measures. The study aims to evaluate the feasibility of integrating the developed ePRO-based system for palliative care in children with cancer into paediatric oncology care and assessing its benefit and support for the child and their parents .

Objectives and Outcome Measures

Primary Objective

The main objective is to assess the feasibility of a comprehensive, patient-centred service for palliative care in children with cancer by adapting and advancing ePRO systems ideally leading to its acceptability and engagement. This will be measured by recording recruitment rate, participation rate, premature discontinuation rate and adherence rate to the different aspects of the MyPal digital health platform.

For peer review only - http://bmjopen.bmj.com/site/about/guidelines.xhtml

Secondary Objectives

A secondary objective is to determine the usage and evaluation of the MyPal apps by the users, including the digital assessment tools for symptom burden in children with cancer. For this purpose, quantitative data is collected from parents through the standardised System Usability Scale (SUS) [10] as well as from the children through a newly adapted version of the SUS. Moreover, qualitative data is collected through interviews and focus groups. This aims to identify further barriers, facilitators and preferences with regard to the MyPal digital health platform to ideally contribute to the evidence base of clinical ePRO use in paediatric oncology and palliative care.

As another secondary objective, the study aims to demonstrate the appropriateness and acceptability of assessment tools

- of children's symptom burden through a novel digital adaptation of the validated print versions of the Mini-SSPedi and SSPedi questionnaires [11, 12].
- of children's QoL under cancer treatment through the PedsQL™ Cancer Module [13, 14].
- of parents' burden through the Impact on Family Scale, specifically with regards to: financial impact, family-social impact and personal strain for the primary family carer [15].
- of the QoL of parents having a child with cancer to be assessed through the EQ-5D-3L [16]. The parents' QoL will be evaluated with regards to the following dimensions: mobility, self-care, usual activities, pain/discomfort and anxiety/depression.
- of parents' satisfaction with the care offered to their children with cancer using the EORTC PATSAT C33 [17, 18] which assesses patients' perception of the quality of medical and nursing care, as well as the organisation of care and services of an oncology department. It has been adapted appropriately to assess parents' satisfaction with children's cancer care in agreement with the authors of the questionnaire.

A final secondary objective is to determine the impact on healthcare professionals in two European countries due to the integration of ePROs in palliative care. This will be measured by a specifically developed web-based online questionnaire evaluating strain parameters such as additional time spent on care, usability, user-experience etc.

Patient Recruitment

In total, 100 children with leukaemia or solid tumours and their parents will be prospectively enrolled in the study across all 3 participating clinical sites:

1. University of Saarland (Germany)

For peer review only

2. Hannover Medical School (Germany)
3. University Hospital Brno (Czech Republic)

At each clinical study site, participants will be screened for eligibility and contacted by the local study team (see Box 1 for eligibility criteria). Information sheets will be handed to the parents and their child (see supplementary appendix 1). For each eligible participant, informed consent will be sought prior to any study-related activities (see supplementary appendix 2). As far as paediatric patients are concerned, information sheets and consent forms have been designed according to different age groups included in the study. Parents and children will be given an appropriate time of at least 24 hours to consider participation and to ask follow-up questions.

BOX 1 Eligibility Criteria

Inclusion Criteria for Children

- 6-17 years of age
- Diagnosed with paediatric leukaemia or solid cancer in the past 12 months
- Receiving anti-cancer treatment at one of the participating clinical sites
- Have age-appropriate speaking, reading and comprehension skills in either the German or the Czech language
- Access to an internet connection and mobile device (e.g. smartphone or tablet)

Inclusion Criteria for Parents

- Parent(s) with a child eligible for the study, as per the inclusion and exclusion-criteria
- Ability to speak, read and understand German or Czech language
- Access to an internet connection and mobile device (e.g. smartphone or tablet)

Exclusion Criteria for Children and Parents

- Anyone who is not able to participate in the study according to the clinical judgment of the site chief investigator or any other authorised person of the research team. This judgment has to be documented for each child/parent not being enrolled.

The patients' and parents' written consent constitutes the start of study enrolment. During the 6 months of study enrolment, children will be asked to complete questionnaires implemented as ePROs and integrated into the serious game via the MyPal platform both at baseline and at regular time intervals after receiving an introductory session by a research team member and in-app tutorials (cf.

For peer review only

Figure 1). Parents as well as HCPs can help the children and complete the questionnaires as proxies throughout the study. Parents will also complete periodic questionnaires at baseline and subsequently using an app provided by the MyPal digital health platform. Independently, the medical care of the child will continue as required throughout and after the course of the study, e.g. anticancer treatment, receiving palliative and/or supportive care. After 6 months of study enrolment, children will be offered to continue playing the game without further data reporting or questionnaires via the MyPal digital health platform. Likewise, data will no longer be collected from their parents. Seven days after the end of data reporting, paper-based follow-up surveys will be completed by the children and their parents to evaluate their judgment on the MyPal platform and its usability.

HCPs will be asked to evaluate the impact and usability of the MyPal platform by a web-based online questionnaire to be completed at the end of the study or earlier if circumstances, e.g. leaving a job, suggest it.

MyPal Digital Health Platform

The MyPal digital health platform and the usage of its applications is the central innovation introduced and tested for feasibility and acceptability in this study. A participatory design was adopted in the development of the digital health platform involving a series of focus groups and discussions with patients and parents in order to identify needs and preferences as well as to validate tools, assess user experience etc. The usage of the MyPal platform revolves around the reporting of physical symptoms caused by the disease and medication as well as QoL of the child with cancer via the MyPal Child app. Secondarily it comprises the reporting of their parents or legal guardians via the MyPal Carer app. The types of users of the MyPal digital health platform are specified in Table 1 while the software and hardware modules of the system are presented in Figure 2.

Table 1: The primary and secondary users of the MyPal digital health platform

User type	Description
Children with cancer	The child cancer patients registered at the participating clinical centres (primary users)
Parents of children with cancer	The parents or legal guardians of the primary users (secondary users)
Healthcare professionals (HCPs)	The interdisciplinary team of treating clinicians (oncologists, haematologists) nurses, psychologists, social workers, other palliative care members) of participating clinical centres (secondary users)

For peer review only

Other parameters (such as satisfaction with care, impact of disease on family, parents' QoL) are also reported by the parents via the MyPal Carer app. The symptom-related information is delivered instantly to the account of the treating HCP and can be visualised within a MyPal web-based application. The usage of the MyPal functionalities is described in more detail below from the standpoint of the patient, the parent and the HCPs.

Children's involvement

The game is designed for a target group from 6 to 17 years old, requiring basic reading skills. Designed as a runner game, the game character is continuously running being controlled by the player through a game environment with collectable items and obstacles. The art style is defined by a colourful underwater exploration (see Figure 3), and specifically designed to appeal to a wide age range. Factors for long- and short-term motivation, e.g. collectibles and rewards, have been incorporated into the game.

Registration Phase. Registration requires actions both in the MyPal Child app and the MyPal web-based app by the HCP. During this phase (1) the patient is registered into the MyPal digital health platform by an HCP or research team member, e.g. research nurse, who also helps with the initial login; (2) a number of preferences are set; (3) baseline assessment of the patient's physical symptoms are collected. In the MyPal Child app, the patient receives a short training session and an in-app tutorial helps to get familiar with the functionalities.

Main usage phase. The main usage phase lasts for 6 months, during which the patient is given access to all the functionalities of the MyPal gamified app, outlined in Table 2. The first three functionalities can be accessed by the patient at any point in time, while the last is activated/deactivated automatically within the MyPal digital health platform on a monthly basis.

Table 2: The functionalities of the MyPal Child app offered to the child patients

	Functionality	Description
**P1**	Game playing	This is a prominent functionality of the MyPal gamified app. The patient is offered to play 3 new run sessions in a day, during which he/she controls a diver swimming in an underwater world. The patient can also customise

For peer review only

		various elements of the game (e.g., the appearance of the diver) at any time.
**P2**	In-game symptom reporting	This is the main ePRO data collection functionality of the MyPal gamified tablet app. During each gaming session, the game pauses 5 times for the user to answer a single symptom-related question (see Figure 3); each question is seamlessly integrated in the flow of the game (the patient still sees the diver in his/her underwater environment). The set of questions to be asked stem from the unique digital adaption based on the validated SSPedi/Mini SSPedi paper-version questionnaire. Each question to be asked during a game session is determined by an internally validated novel question prioritisation algorithm.
**P3**	Spontaneous symptom reporting	This is the secondary functionality that allows patients to report physical symptoms whenever they wish. An electronic implementation of the SSPedi/Mini SSPedi questionnaire outside the game is employed for the spontaneous reporting.
**P4**	Periodic QoL reporting	This is the secondary functionality that asks patients to report their QoL once per month. An electronic implementation of the validated PedsQL questionnaire is therefore employed for the spontaneous reporting. An optional read-aloud function for questions can be turned on to support especially young children.

Parent's involvement

The parents of children with cancer interact with the MyPal reporting smartphone app in 2 sequential phases, which are outlined below.

Registration Phase. During this phase (1) the parent is linked to their child within the MyPal digital health platform; (2) baseline assessment of a subset of the outcome measures of the study via self-reporting are collected. Parents undergo a short training session and are guided during registration by an HCP participating in the study, e.g. research nurse.

Main usage phase. The main usage phase lasts for 6 months and is aligned with the patient participation in the study. The only functionality that is offered to the parent is reporting, either as a

For peer review only - http://bmjopen.bmj.com/site/about/guidelines.xhtml

proxy or for themselves, concerning a subset of the outcome-measures of the study. The reporting functionality of the MyPal Carer app is outlined in Table 3.

Table 3: The functionalities of the MyPal Carer app offered to the child's parents

	Functionality	Description
**R1**	Patient QoL reporting	Proxy reporting of the QoL for the patient using an electronic implementation of the validated PedsQL questionnaire. Older patients will be asked to complete the questionnaire directly in the MyPal Child app. This is linked with an outcome measure of the study and is reported once per month.
**R2**	Parent QoL reporting	Reporting of the QoL of the parent using an electronic implementation of the validated EQ-5D-3L questionnaire. This is linked with an outcome measure of the study and is reported once per month.
**R3**	Satisfaction with care reporting	Reporting of the satisfaction with received care using an electronic implementation of the validated PATSAT-C33 questionnaire. This is linked with an outcome measure of the study and is reported once per month.
**R4**	Disease impact on family scale reporting	Reporting of the impact of the disease on the family using an electronic implementation of the validated Impact on Family Scale questionnaire. This is linked with an outcome measure of the study and is reported once per month.
**R5**	Patient Symptom Reporting	Proxy reporting of symptoms for the patient using an electronic adaption of the SSPedi/Mini-SSPedi questionnaire. Usually patients will be asked to answer questions with regard to symptoms directly in the gamified app.

HCP's involvement

Registration Phase. After a short registration procedure that takes place during their first visit to the web-based app, the HCPs can continuously interact with it throughout the duration of the study. HCPs are given a short in-app training session.

Main usage phase. During the main usage of the MyPal web-based app, the HCPs can access symptom related data being stored in the system backend which had been collected by (1) the MyPal Child app and (2) the MyPal Carer app. HCPs are authorised to solely access the data of the patients of their associated clinical centre. The individual data of participating patients are reviewed by the associated HCP at least once every 72 hours to check for new entries. The data review and any action related to this are recorded through the web interface. The functionalities of the MyPal web-based app are outlined in Table 4. All of them can be accessed by the HCP at any time. In addition, the HCP can use the MyPal Carer app in order to complete questionnaires as a proxy for one of their patients.

Table 4: The functionalities of the MyPal web-based app offered to the HCPs

	Functionality	Description
**H1**	Incoming information summary	A central page of the web-based app displays in a summarised form the incoming patient information that has not been reviewed yet. Incoming information is automatically prioritised in the system using custom algorithms whereby pieces of incoming information are assigned the highest priority and placed at the top of the list. Whenever an item is reviewed in full, it is removed from the list.
**H2**	Individual data dashboard	A page that presents all the information collected for a given patient since the start of their participation in the study, using a dashboard with modern visualisations (cf. Figure 4). The information includes primarily the patient's responses to the symptom questionnaire along with additional clinical information (e.g., treatment, age group, diagnosis group etc.).
**H3**	Symptom Status over Time	This functionality and its visualisation are linked to the individual data dashboard. HCPs can document interim reports on current or past symptoms at different points in time as well as corresponding measures taken via selection of pre-defined choices. Additionally, the current or retrospective status of the patient's disease as well as the goal of treatment can be updated considering the whole course of the study.

**H4**	Aggregated data dashboard	A page that presents aggregated and summarised information coming from all patients that participate in the study (descriptive statistics such a min, max, average and percentiles) using an analytics dashboard with modern visualisations.
**H5**	HCP response log	A page that is used for logging potential responses (e.g., referral to a specialist or prescription of medical examinations) of the HCP to the presented information of a specific patient. The HCP can log any actions taken after visiting individual data dashboard of a patient in a structured manner.

peer review only

Data Collection and Analysis

All applicable national and EU legislation, particularly the General Data Protection Regulation (EU) 2016/679 [19] for the protection of individuals have been considered in the design of the study-related IT infrastructure with regard to the confidential processing, collection and access to personal data. The technical deployment of the MyPal platform comprises of local server installations at each of the clinical study sites and one central server installation at the sponsor's site distinctly defining premises of data accessibility. The data security concept entails regular synchronisation of only anonymised data from local to central database. The apps being installed on mobile devices are protected by confidential credentials and store encrypted data only temporarily locally in case of absent internet connection until they are deleted from the mobile device subsequent to transmission to the respective databases.

Sample size calculation

Assuming relatively acceptable values for the attrition rate (i.e., 20%) and the missing data (i.e., 30%), the sample size analysis concluded that 100 recruited paediatric patients providing one measure at enrolment (baseline) and 6 repeated measures (at Months 1, 2, 3, 4, 5, 6) are sufficient for the power of the intended statistical testing to be over 90% in all cases, given (a) a 0.05 significance level, and (b) an effect size of 0.1; the employed value of the effect size was based on a priori knowledge of the domain, all power calculations were performed using the G*Power statistical analysis software [20].

Data Analysis

The subsequent analysis and evaluation will serve to assess the feasibility of the MyPal digital healthcare platform which uses ePROs to be ideally beneficial and supportive for the child and parents in palliative care.

Subgroup analysis of the outcome measures will be performed at baseline, Month 3 and Month 6 of the study using one-, two- and three-way ANOVA in order to detect potential differences between specific groups of participants. The level of significance for all statistical tests is set to a=0.05, in accordance with the power calculations. The grouping variables that will be assessed are (a) the clinical centre (origin), (b) the country of residence, (c) the age group, (d) the disease category (leukaemia, solid tumour, brain tumour), (e) the disease stage and (f) the starting point of the study in relation to the date of diagnosis. The latter point refers to recruited patients who have been under treatment for more than six months receiving care at the respective participating clinical site. These patients constitute a cohort for retrospective baseline data collection: the data collected from their completion of the ePROs at the beginning of their study enrolment will later be compared to outcome measure data derived from prospectively recruited patients whose treatment started within the last 6 months. Both groups will be followed likewise during the course of the study. Further data, like the daily number of steps (estimated by the smartphone/tablet using the measurements of the built-in sensors) as well as the documentation of the symptom status over time, will be used to examine the feasibility to assess for correlations with reported symptoms and reported physical activity.

To evaluate the changes in outcome measures over time in the child cohort, repeated measures analysis of variance (ANOVA) will be applied on each measure (or a non-parametric equivalent). Post-hoc analysis will be applied as appropriate. Since wording and layout of questionnaires were partly modified compared to the validated versions for electronic completion and gamification, another focus is (or would be) to assess the feasibility of such modifications to the original questionnaires. Repeated measures multivariate ANOVA (MANOVA) will be applied to the QoL of the children and the parents which serve as a pair of dependent variables.

To examine potential QoL differences between the participating sites, independent-samples t tests and analysis of covariance (ANCOVA) models will be applied that will control for baseline criterion scores and potential confounders such as age group and sex which may be imbalanced between groups and associated with outcomes of interest.

The potential effect of the attrition is going to be assessed according to the methods introduced in Fewtrell *et al*. [21], e.g., comparison of baseline characteristics of seen versus not seen at the follow-

For peer review only

up participants to assess potential bias, sensitivity analysis, re-evaluation of the power of the study with the attained sample size. The impact of missing data in the attempted analysis is going to be evaluated based on the importance of the variables. Checks for data “missing at random” and “missing completely at random” are going to be performed.

Additional quantitative feedback will be gathered by a final evaluation of the MyPal digital health platform based on an appropriate usability one-time scale, i.e. system usability scale for patients or parents, whereas HCPs’ feedback will be collected via a designed web-based questionnaire at the end of the study.

Concluding Remarks

The foreseen advancement of the presented MyPal’s patient-centred ePRO approach will offer a significant opportunity for children with cancer, their parents and healthcare providers to actively participate in the care process. By exploiting technological advances in information and communication technologies, MyPal aspires to contribute to bridge the gap between timely reporting and tracking of symptoms and the personalised actions performed by healthcare providers addressing the patient’s needs which can vary across the disease course. The paradigm shift from passive patient reporting to active patient engagement could both enhance palliative and supportive care for children with cancer and improve coping with the disease.

Author Contributions

MM, NG, CK, CM, AS, SP, LS, PL drafted the study protocol
MM and CK contributed equally as main authors on the manuscript
NG, TG, SP, AS, JD, CM, KS, JL, LS, CP contributed to critical revisions of the study protocol and the manuscript.
NG, AS and PL are the Site Chief Investigators and take overall responsibility for all aspects of the study design, the protocol and the study conduct at the involved clinical study sites.

Acknowledgments

MyPal is coordinated by Dr Kostas Stamatopoulos of the Centre for Research and Technology Hellas, Greece. Other partners are: Fraunhofer Institute for Biomedical Engineering, Germany; Foundation for Research and Technology Hellas, Greece; International Observatory on End of Life Care, Lancaster University, UK; Central European Institute of Technology, Masaryk University, Czech Republic; Karolinska Institute, Sweden; Vita-Salute San Raffaele University, Italy; University Hospital of Heraklion, Greece; Hannover Medical School, Germany; University Hospital Brno, Czech Republic;

Saarland University, Germany; Promotion Software GMBH, Germany; Atlantis Healthcare, UK; European Association for Palliative Care; International Children's Palliative Care Network; National School of Public Health, Greece. The authors are grateful to the clinicians at the clinical study sites who are essential to this study (patient recruitment, symptom assessment and management and data collection). This work is dedicated to Vassilis Koutkias.

Funding

MyPal: Fostering Palliative Care of Adults and Children with Cancer through Advanced Patient Reported Outcome Systems, is funded by the Horizon 2020 Framework Programme of the European Union under Grant Agreement Nr. 825872.

Competing interests

None declared.

Ethics approval

The MyPal-Child study protocol has received ethical approval from the Ethics Committee competent for the University of Saarland: Ärztekammer des Saarlandes (Ha 23/20). Ethics approval has also been received from all of the following: Ethics Committee of the Medical School Hannover (9095_BO_K_2020) as well as the Ethics Committee of the University of Brno (01-120220/EK).

Provenance and peer review

Not commissioned; externally reviewed.

Figure Legends

Figure 1: Patient-specific study enrolment schema of MyPal4Kids.

Figure 2: Software and hardware modules of the MyPal digital health platform. The MyPal Child and Carer app are useable via various types of mobile devices. The user of the MyPal Carer app are the parents/legal guardians involved in the study. Healthcare professionals can use the MyPal Carer app as well to report as proxy for the child.

Figure 3: A) A screenshot of the gamified symptom questionnaire as part of the MyPal Child app. B) Screenshots taken of the MyPal Carer app depicting the PATSAT C-33 questionnaire with instruction screen, selection of patient setting screen and one sample question screen.

Figure 4: Symptoms reported by study participants can be visualised in the MyPal web-based app for HCPs to be reviewed according to adjustable settings.

References

[1] World Health Organization. (1998). Cancer pain relief and palliative care in children. World Health Organization

For peer review only

[2] Hoerger M, et al. Defining the Elements of Early Palliative Care That Are Associated With Patient-Reported Outcomes and the Delivery of End-of-Life Care. J Clin Oncol. 2018;36(11):1096-102.

[3] Leahy AB, Feudtner C, Basch E. Symptom monitoring in pediatric oncology using patient-reported outcomes: why, how, and where next. The Patient-Patient-Centered Outcomes Research. 2018 Apr 1;11(2):147-53.

[4] US Food and Drug Administration. Guidance for industry: patient-reported outcome measures: use in medical product development to support labelling claims: draft guidance. Health Qual Life Outcomes. 2006;4:79.

[5] Eliasen A, Abildtoft MK, Krogh NS, Rechnitzer C, Brok JS, Mathiasen R, Schmiegelow K, Dalhoff KP Smartphone App to Self-Monitor Nausea During Pediatric Chemotherapy Treatment: User-Centered Design Process. JMIR Mhealth Uhealth 2020;8(7):e18564

[6] LeBlanc TW, Abernethy AP. Patient-reported outcomes in cancer care - hearing the patient voice at greater volume. Nat Rev Clin Oncol. 2017;14:763-72.

[7] Downing J, Namisango E, Harding R. (2018) Outcome measurement in paediatric palliative care: lessons from the past and future developments. Annals of Palliative Medicine. doi: 10.21037/apm.2018.04.02

[8] Bausewein C, Daveson BA, Currow DC, Downing J, Delians L, Radbruch L, Defilippi K, Lopes Ferreira P, Costanini M, Harding R and Higginso IJ. (2015) EAPC White Paper on outcome measurement in palliative care: Improving practice, attaining outcomes and delivering quality services – Recommendations from the European Association for Palliative Care (EAPC) Task Force on Outcome Measurement. Palliative Medicine.

[9] Maguire R, et al. The eSMART study protocol: a randomised controlled trial to evaluate electronic symptom management using the advanced symptom management system (ASyMS) remote technology for patients with cancer.BMJ Open. 2017;7(5):e015016.

[10] Peres SC, Pham T, Phillips R. Validation of the System Usability Scale (SUS). In: Proceedings of the Human Factors and Ergonomics Society Annual Meeting, 2013; 57(1):192–196.

[11] Tomlinson, Deborah; Hyslop, Shannon; Stein, Eliana; Spiegler, Brenda; Vettese, Emily; Kuczynski, Susan et al. Development of mini-SSPedi for children 4-7 years of age receiving cancer treatments. In: BMC cancer; 2019; 19(1):32.

[12] Dupuis LL, Johnston DL, Baggott C, Hyslop S, Tomlinson, D, Gibson P, et al. Validation of the Symptom Screening in Pediatrics Tool in Children Receiving Cancer Treatments. In: Journal of the National Cancer Institute 2018; 110(6): 661–668.

For peer review only

[13] Varni JW, Burwinkle TM, Katz ER, Meeske K, & Dickinson P. The PedsQL in pediatric cancer: Reliability and validity of the Pediatric Quality of Life Inventory Generic Core Scales, Multidimensional Fatigue Scale, and Cancer Module. Cancer, 2002; 94:2090-2106

[14] Robert RS, Paxton RJ, Palla SL, Yang G, Askins MA, Joy SE, Ater JL. Feasibility, reliability, and validity of the pediatric quality of life inventory™ generic core scales, cancer module, and multidimensional fatigue scale in long-term adult survivors of pediatric cancer. Pediatric Blood & Cancer 2012; 59:703–707.

[15] Stein REK & Riessman CK. The development of the Impact on Family Scale: Preliminary findings. Medical Care, 1980; 18.465-472.

[16] EuroQol Research Foundation. EQ-5D-3L User Guide, 2018. Available from: https://euroqol.org/publications/user-guides. Last accessed: October 1, 2019.

[17] Bredart A et al. "Phase III study of the European Organisation for Research and Treatment of Cancer satisfaction with cancer care core questionnaire (EORTC PATSAT-C33) and specific complementary outpatient module (EORTC OUT-PATSAT7)." European Journal of Cancer Care (English Language Edition); 2018; 27(1).

[18] Bredart A et al. "The European organization for research and treatment of cancer – satisfaction with cancer care questionnaire: revision and extended application development." Psycho-Oncology 2017; 26(3): 400-404.

[19] Regulation (EU) 2016/679 of the European Parliament and of the Council of 27 April 2016 on the protection of natural persons with regard to the processing of personal data and on the free movement of such data, and repealing Directive 95/46/EC (General Data Protection Regulation) (Text with EEA relevance) Official Journal L119, 4.5.2016:1–88.

[20] Faul F, Erdfelder E, Lang AG, & Buchner A. G*Power 3: A flexible statistical power analysis program for the social, behavioral, and biomedical sciences. Behavior Research Methods, 39. 2007;175-191

[21] Fewtrell MS, Kennedy K, Singhal A, Martin RM, Ness A, Hadders-Algra M, Koletzko B, Lucas A. How much loss to follow-up is acceptable in long-term randomised trials and prospective studies?. Archives of Disease in Childhood. 2008;93(6):458-61.

For peer review only

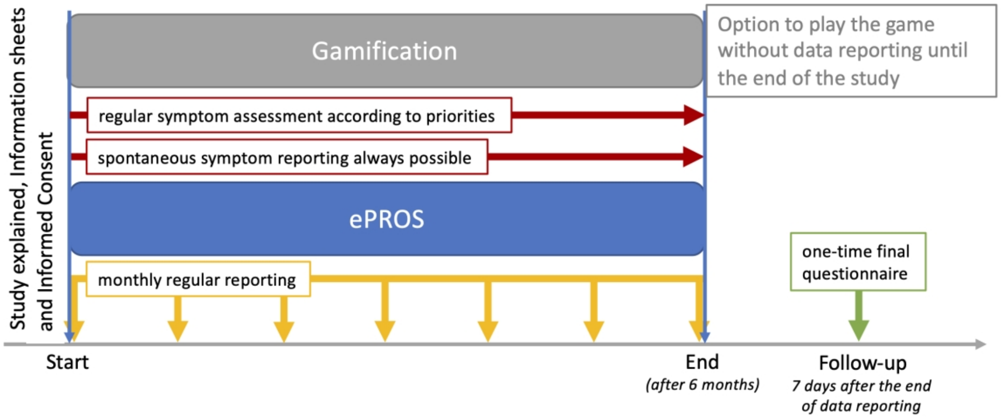

Patient-specific study enrolment schema of MyPal4Kids.

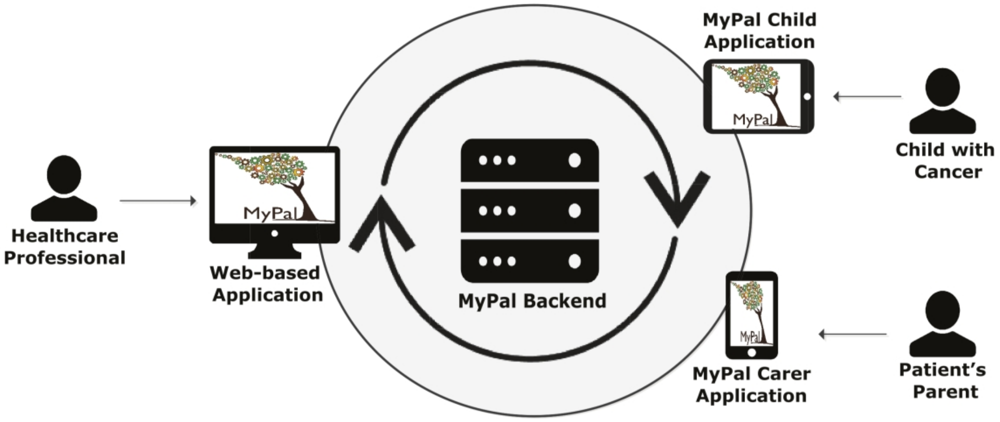

Software and hardware modules of the MyPal digital health platform. The MyPal Child and Carer app are useable via various types of mobile devices. The user of the MyPal Carer app are the parents/legal guardians involved in the study. Healthcare professionals can use the MyPal Carer app as well to report as proxy for the child.

239x101mm (300 x 300 DPI)

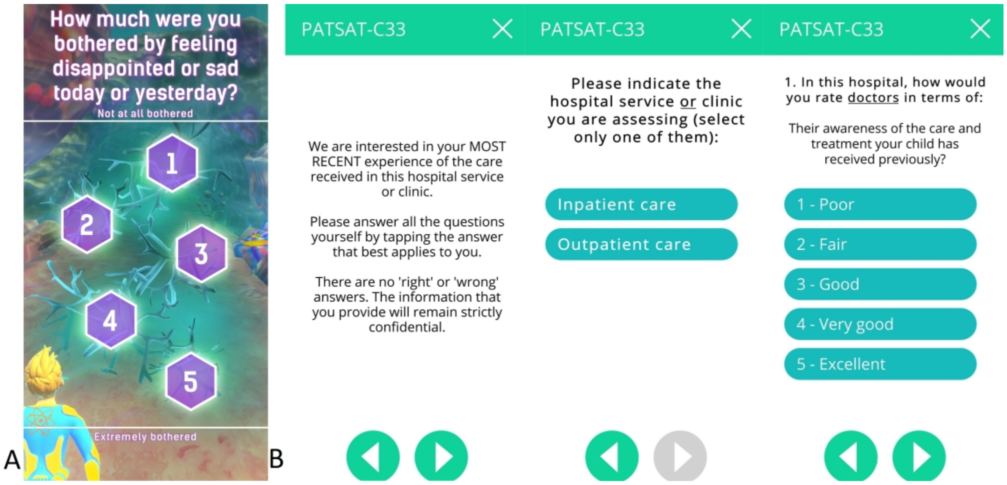

A) A screenshot of the gamified symptom questionnaire as part of the MyPal Child app. B) Screenshots taken of the MyPal Carer app depicting the PATSAT C-33 questionnaire with instruction screen, selection of patient setting screen and one sample question screen.

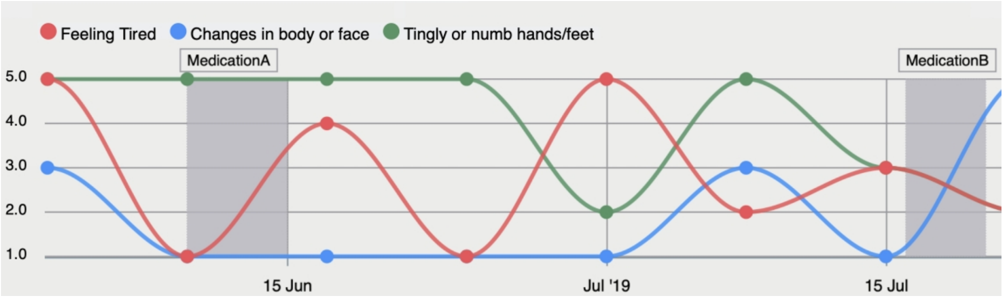

Symptoms reported by study participants can be visualised in the MyPal web-based app for HCPs to be reviewed according to adjustable settings.

Version 1.1 – 05.03.2020

Informed Consent Form for Parents / Legal Guardian

- ☐ I consent to use Internet connected devices, e.g. smart phone or tablet, to complete self-reporting questionnaires monthly via an app to assess well-being, satisfaction with cancer care received for my child and their impact on the family.
- ☐ I consent to respond one-time to a paper-based questionnaire 7 days after the 6 months of study enrollment to evaluate the usability of the MyPal platform.
- ☐ I consent that I have read and understand the information sheet explaining the above research project and I have had the opportunity to ask questions about it.
- ☐ I consent that information has been provided to me in a language that I fully understand.
- ☐ I am aware that NO biological samples will be collected from my child in the study and that the study does NOT entail the administration of any medication to my child.
- ☐ I am aware that the participation of my child is absolutely voluntary and that I am free to withdraw my consent at any time without giving any reason and without there being any negative consequences.
- ☐ I consent that if I withdraw myself or my child from the study after some data have been collected, these data will be anonymized but not deleted. No further data will be collected in this case.
- ☐ I am aware that in all research outputs (papers, presentations, articles, reports) the anonymity the data will be protected.
- ☐ I am aware that data collected during MyPal project will never be transferred to countries outside of the European Union.
- ☐ I consent that medical data that has been obtained during this project will be stored for 15 years, in (name of the clinical site) and the data that has been collected for the purposes of the study, will be kept in a de-identified form at the central installation of the sponsor of the study.
- ☐ I consent that health care professionals will report disease-related data of your child to the MyPal system.
- ☐ I am aware that any supportive information provided by health care professionals (e.g. physicians) via the MyPal platform does not imply legal liability and that MyPal is not designed as an emergency alert system and doctors might not respond immediately.
- ☐ I consent that I have understood the information sheet for the study including information on rights for affected person in terms of personal data. Hereby, I have obtained the contact details of the Data Protection Officer responsible, in case I have concerns or queries explicitly dealing with data protection or want to lodge a complaint due to this issue.

I have read and understood this consent form and I have been provided with information regarding the research study. I have been given a copy of the information sheet and of this consent form.

In case I have concerns of any kind or further questions, I may contact (enter contact details)

A copy of this agreement will be given to me and another copy will be retained for record keeping by the project.

○ **I agree for my child to participate in the study**

Name of the child: ______________________________________, Birthday: ___.___.20….

________________	________	________________
Name of the Mother	Date	Signature
Name of the Father	Date	Signature
Name of the Researcher	Date	Signature

For peer review only - http://bmjopen.bmj.com/site/about/guidelines.xhtml

Version 1.1 – 05.03.2020

Clinical Study MyPal 4KIDS

MyPal-Child Information Sheet for Parents / Legal Guardian

We would like to invite your child to participate in a study conducted by Dr (enter authorized clinician) and his/her team at the University of (enter Clinical Site). Before you decide to give permission for your child, we would like to help you understand the study and what is involved. Please take time to read the following information carefully and to decide whether or not you wish your child to take part.

In this study we are interested in exploring the use of digital technology that will help you (and possibly members of your family) to communicate your condition more accurately and effectively to your health care providers (i.e. oncologists, specialized physicians, psychologists, nurses). The aim is to improve the quality of care by using modern methods of individualized information, communication and support for patients with cancer the main focus of which is the patient (Patient-Reported Outcome). Supportive information provided by health care professionals via the application do not imply legal liability.

Aims of the study: The study in the context of the EU project MyPal[1] aims to propose a new system of care that is anticipated to improve quality of life in children with cancer and their parents, by exploring how digital technology can enhance communication, improve decision-making, emotional support and reinforcement of young patients and reduce symptom burden. It only involves observation of your child regarding the use of an application in a gamified form. It is important for you to know that the study:

- does not involve collection of biological samples
- does not involve administration of any medication
- does not aim to provide or change medical treatment solely based on the MyPal platform
- MyPal is not an emergency alert system and doctors may not respond immediately

Why has your child been chosen: Your child has been chosen to participate in this study because he /she is between 6-17 years of age and has been diagnosed with acute leukemia or solid cancer for which anti-cancer treatment is already being provided. Also, because he/she is fit to participate in the study and able to use an Internet connected device (smartphone or tablet). The contribution of children and adolescents is very important for the study to explore their specific needs and preferences and with the aim of making young people with cancer stronger to deal with their condition.

What does your child have to do: If you and your child agree to participate, your child will receive an introduction to get familiar with the MyPal App and its tools and how to use them. For the upcoming 6 months, your child can then perform the following tasks:

Use the MyPal app through Internet connected devices, such as smart phone, tablet for a game which involves questionnaires about their well-being and symptoms. In addition, the physical activity of your child will be roughly estimated based on a daily step counter.
With regard to the various questionnaires involved, the child will be free to choose not to answer any particular question or questions. Completion will be scheduled at baseline, and then monthly, some of them several times a week, until the end of the study. It is possible that these time intervals may change due to the needs of the study.
Respond only once to paper-based questionnaire 7 days following the 6 months of study enrollment in order to evaluate the usability of the MyPal platform.

During the completion of the questionnaires your child may get assistance by you, a member of our research team or a health care professional.

Which tasks you as parents/legal guardian will be asked to complete:

You will also receive an introduction on how to get familiar with and use the MyPal platform and its tools.
Use the MyPal platform to complete monthly questionnaires about your well-being, satisfaction with your child's received care and the impact of the disease on you and your family.
Help your child completing questionnaires if they ask you for help.
respond only once to a paper-based questionnaire 7 days following the 6 months of study enrollment in order to evaluate the usability of the MyPal platform.

[1] Available from: https://mypal-project.eu/. Last accessed: January 20, 2019

For peer review only - http://bmjopen.bmj.com/site/about/guidelines.xhtml

Version 1.1 – 05.03.2020

Will the data collected in this project be kept confidential? Data protection is one of our most important priorities in this study. National laws on personal data protection will be implemented in order to guarantee the highest standards in personal data management. Further, all procedures for protecting personal information in this study are in accordance with the approved rules of the University of (enter clinical site) and with the European legislation including the General Data Protection Regulation 2016/679 (GDPR). Only data that is necessary for this research and no additional data will be collected. Our technology partners will provide technical support and tools such that data protection and security requirements are ensured. Any information, that is obtained in this study and that can be assigned to you or your child will remain confidential.

If you agree for your child to take part in this study, we will use his/her data in the ways needed to conduct our study and analyse the study results. Your rights to access, change or move your child's information are limited, as we need to manage this information in specific ways in order for the research to be reliable and accurate (cf. point d of Article 17(3) GDPR and Article 89 GDPR). If your child withdraws from the study, we will keep the information that we have already obtained and anonymize it for analysis at the end of the study. Anonymization means that the stored data is altered in such a way that it cannot be restored in its original form and all information which could link the collected data to you or your child is deleted. No more data will be collected after withdrawal.

We will not allow people from outside the project to know how you or your child answered the questions within the study. In case you have concerns or queries explicitly concerning data protection or you want to get into contact or lodge a complaint with data protection authorities, you can also contact the Data Protection Officer of our Research Institution: the corresponding contact details can be found on the last page.

Storage of data and security: Data will be stored in two ways, locally and centrally, which is elucidated in the following.

The app installed on the mobile device stores encrypted data, i.e. answers to questionnaires, only temporarily locally if the mobile device has no internet connection. As soon as an internet connection is available, the answers will be transmitted to the local database at your hospital and deleted from the mobile device. After the initial login to the app, an access token is stored on the device which facilitates each upcoming login.

The local database of (name of the clinical site) will store:

- responses to questionnaires about symptoms, spontaneous symptom reporting forms, etc.
- personal data (patient name, provenance, treating clinical site)
- clinical information (gender, age, diagnosis, disease stage/risk, treatment scheme, expected outcome, functional impairment, etc.). Concerning the clinical information, the (a) age and (b) diagnosis of the study subject are categorized before storage instead of precise detail.

The data included in the local database will be accessible only by the team of local investigators, and even the members of the team will only have access to the information needed for the specific user category with regard to the study. All personally identifiable data remain at any time at the (enter clinical site) and will neither be shared with the other participating clinical sites nor study sites. Furthermore, the data is protected against access by unauthorized people by username and password.

The central database which resides at the side of the sponsor of the study (Centre for Research and & Technology Hellas, Greece) and which fulfills all technical and organizational requirements for the safety and the security of the stored data to protect them against unauthorized people's access by access restriction. The central database will include a part of the local data (responses to the questionnaires and information needed to assess the endpoints of the study, e.g. quality of life, satisfaction with care etc.) which will be transferred from the participating clinical center to the central database identification to enable the joint analysis of data from the participating clinical sites. Before the transmission, personal data is removed (Anonymization). Anonymization is performed by removing the patient's name from the personal data and keeping among the clinical data only the gender, age group and categorized diagnosis information.

For peer review only - http://bmjopen.bmj.com/site/about/guidelines.xhtml

Version 1.1 – 05.03.2020

All data obtained in the study will be maintained for 15 years in the (enter name of the clinical site) where they have been collected or created.

Does your child have to participate? Participation is completely voluntary and you are free to consent or not to the participation of your child. Even if you agree, you remain free to withdraw this consent at any time without giving any reason and without this affecting your or the child's relationship with our research team or with the team providing clinical care. If you decide to withdraw from the study once data collection commenced, the collected data will not be erased, but will be retained in the study but in an anonymized form which will not in any way permit the child's or your identification. Once the research has been completed and the data been analysed, it will not be possible to withdraw this data from the study. No more data will be collected from you or your child after the withdrawal.

Are there possible disadvantages and/or risks in taking part? Participation of your child does not entail any risk of discomfort, pain, injury, illness or disease and we remind you that the study itself does NOT involve collection of biological samples or administration of any medication. However, if we come across an unexpected finding which we think requires notifying you, we plan to inform you, to discuss this in our team, and to consult with your child's treating physician. If you have any concerns please feel free to contact the Principal Investigator (see below for details) and once again, we would like to remind you that your participation is entirely voluntary. The health care professionals do not bear liability for information which are provided to the patients or parents via the Apps.

What are the possible benefits of taking part? You, your child, or your family may not receive any personal benefits from participating in the study except from the possibility to use and play the game. However, you may find participation in the study is a positive experience, through the use of the apps and by contributing to the improvement of the quality of life of young cancer patients in the process of their treatment.

Transfer of data: All data collected during the MyPal project will not be transferred to countries outside of the European Union.

What will happen to the results of the research project? The results will be used only for research purposes; they may be reported in research publications and may be made available to other researchers in an anonymized form, e.g. in presentations. In every research output (papers, presentations, articles, reports) the complete anonymity of your data will be ensured.

Right of the individual affected in terms of processing personal data

Based on the EU Data Protection Basic Regulation 2016/679 (GDPR), you are entitled the following data protection rights for individuals affected, which you can assert against the involved hospital operators.
You have the right of access to stored personal data relating to you (Article 15 GDPR). If you discover that incorrect data concerning your person is being processed, you can request correction or purpose-related supplementation (Article 16 GDPR).
You have the right to request the deletion of your data if there are specific reasons for deletion. This is particularly the case if they are no longer necessary for the purpose for which they were originally collected or processed (Article 17 GDPR).
You have the right to restrict the processing of your data, which means that your data will not be deleted but will be for data portability (Article 20 GDPR).
In principle, you also have a general right of objection to lawful data processing which is of public interest, in the exercise of official authority or on the basis of the legitimate interest of a site (Article 21 GDPR).
As already mentioned, however, these rights may be restricted in accordance with point d of Article 17(3) GDPR and Article 89 GDPR if they make it impossible or seriously impair the achievement of the study objectives.

Complaint to the supervisory authority in case of data protection violations

You have the right to forward a complain to the supervisory authority if you think that your personal data is being processed unlawfully. The address of the supervisory authority responsible for (specify clinical partner) is:

For peer review only - http://bmjopen.bmj.com/site/about/guidelines.xhtml

Version 1.1 – 05.03.2020

The State Commissioner for Data Protection (give details of the respective State Commissioner for Data Protection + insert address)

Contact with the data protection officer of (specify clinical partners) is as follows:

(please specify address, e-mail, telephone)

The contact to the local study director project at (indicate clinical partner) is as follows:
(indicate corresponding address/email/telephone number WITHOUT mobile phone number)

If you have any questions or concerns regarding the study, please do not hesitate to contact him/her at any time for further information.

Ethical approval: This study has obtained a positive vote by the Ethics Committee of the University of (enter clinical site) which means that the conduction of the study does not give rise to any objections from the ethical point of view.

For peer review only - http://bmjopen.bmj.com/site/about/guidelines.xhtml

BMJ Open

The MyPal-Child study protocol: an observational prospective clinical feasibility study of the MyPal ePRO-based early palliative care digital system in paediatric oncology patients

Journal:	*BMJ Open*
Manuscript ID	bmjopen-2020-045226.R1
Article Type:	Protocol
Date Submitted by the Author:	30-Jan-2021

Complete List of Authors:	Meyerheim, Marcel; Saarland University - Campus Homburg, Clinic of Pediatric Oncology and Hematology<br>Karamanidou, Christina; Centre for Research and Technology-Hellas, Institute of Applied Biosciences<br>Payne, Sheila; Lancaster University, International Observatory on End of Life Care, Institute for Health Research<br>Garani-Papadatos, Tina; University of West Attica, Department of Public Health Policy<br>Sander, Annette; Hannover Medical School, Clinic for Paediatric Oncology and Haematology<br>Downing, Julia; International Children's Palliative Care Network<br>Stamatopoulos, Kostas; Centre for Research and Technology-Hellas, Institute of Applied Biosciences<br>Ling, J.; European Association for Palliative Care, Head Office<br>Payne, Cathy; European Association for Palliative Care, Head Office<br>Scarfò, Lydia; Università Vita Salute San Raffaele, Department of Onco-Haematology, Division of Experimental Oncology<br>Lokaj, Petr; University Hospital Brno, Department of Pediatric Oncology<br>Maramis, Christos; Centre for Research and Technology-Hellas, Institute of Applied Biosciences<br>Graf, Norbert; Saarland University Hospital and Saarland University Faculty of Medicine, Clinic of Pediatric Oncology and Hematology

**Primary Subject Heading**:	Health informatics
Secondary Subject Heading:	Oncology, Palliative care, Paediatrics, Patient-centred medicine
Keywords:	Paediatric oncology < PAEDIATRICS, Paediatric palliative care < PALLIATIVE CARE, Health informatics < BIOTECHNOLOGY & BIOINFORMATICS

SCHOLARONE™
Manuscripts

I, the Submitting Author has the right to grant and does grant on behalf of all authors of the Work (as defined in the below author licence), an exclusive licence and/or a non-exclusive licence for contributions from authors who are: i) UK Crown employees; ii) where BMJ has agreed a CC-BY licence shall apply, and/or iii) in accordance with the terms applicable for US Federal Government officers or employees acting as part of their official duties; on a worldwide, perpetual, irrevocable, royalty-free basis to BMJ Publishing Group Ltd ("BMJ") its licensees and where the relevant Journal is co-owned by BMJ to the co-owners of the Journal, to publish the Work in this journal and any other BMJ products and to exploit all rights, as set out in our licence.

The Submitting Author accepts and understands that any supply made under these terms is made by BMJ to the Submitting Author unless you are acting as an employee on behalf of your employer or a postgraduate student of an affiliated institution which is paying any applicable article publishing charge ("APC") for Open Access articles. Where the Submitting Author wishes to make the Work available on an Open Access basis (and intends to pay the relevant APC), the terms of reuse of such Open Access shall be governed by a Creative Commons licence – details of these licences and which Creative Commons licence will apply to this Work are set out in our licence referred to above.

Other than as permitted in any relevant BMJ Author's Self Archiving Policies, I confirm this Work has not been accepted for publication elsewhere, is not being considered for publication elsewhere and does not duplicate material already published. I confirm all authors consent to publication of this Work and authorise the granting of this licence.

For peer review only

The MyPal-Child study protocol: an observational prospective clinical feasibility study of the MyPal ePRO-based early palliative care digital system in paediatric oncology patients

Marcel Meyerheim[1], Christina Karamanidou[1], Sheila Payne, Tina Garani-Papadatos, Annette Sander, Julia Downing, Kostas Stamatopoulos, Julie Ling, Cathy Payne, Lydia Scarfò, Petr Lokaj, Christos Maramis and Norbert Graf[2]

[1] contributed equally to this paper, [2] corresponding author

Co-first authors

Marcel Meyerheim
Clinic of Pediatric Oncology and Hematology
Saarland University – Campus Homburg
Homburg, Saarland, DE
marcel.meyerheim@uks.eu

Christina Karamanidou
Institute of Applied Biosciences
Centre for Research and Technology-Hellas
Thessaloniki, Central Macedonia, GR
ckaramanidou@certh.gr

Contributing Authors

Sheila Payne
International Observatory on End of Life Care
Institute for Health Research
Lancaster University
Lancaster, Lancashire, UK
s.a.payne@lancaster.ac.uk

Tina Garani-Papadatos
Department of Public Health Policy
University of West Attica
Athens, Attica, GR
sgarani@uniwa.gr

Annette Sander
Clinic for Paediatric Oncology and Haematology
Hannover Medical School
Hannover, Niedersachsen, DE
sander.annette@mh-hannover.de

Julia Downing
International Children's Palliative Care Network
Bristol, Avon, UK
julia.downing@icpcn.org

For peer review only

Kostas Stamatopoulos
Institute of Applied Biosciences
Centre for Research and Technology-Hellas
Thessaloniki, Central Macedonia, GR
kostas.stamatopoulos@certh.gr

Julie Ling
Head Office
European Association for Palliative Care
Vilvoorde, BE
julie.ling@eapcnet.eu

Cathy Payne
Head Office
European Association for Palliative Care
Vilvoorde, BE
cathy.payne@eapcnet.eu

Lydia Scarfò
Department of Onco-Haematology
Division of Experimental Oncology
Universitá Vita Salute San Raffaele
Milano, IT
scarfo.lydia@hsr.it

Petr Lokaj
Department of Pediatric Oncology
University Hospital Brno
Brno, Jihomoravský, CZ
lokaj.petr@fnbrno.cz

Senior Author

Christos Maramis
Institute of Applied Biosciences
Centre for Research and Technology-Hellas
Thessaloniki, Central Macedonia, GR
E-Mail: chmaramis@certh.gr

Corresponding Author and Senior Author

Norbert Graf
Clinic of Pediatric Oncology and Hematology
Saarland University Hospital and Saarland University Faculty of Medicine
Homburg, Saarland, DE
E-Mail: graf@uks.eu

Word Count

3690 words excluding title page, abstract, references, figures and tables

For peer review only

Keywords

- Patient-Reported Outcome (PRO)
- electronic Patient-Reported Outcome (ePRO)
- Patient-Reported Outcome Measure (PROM)
- Palliative Care for Children
- Supportive Care for Children
- Serious Game
- Mobile Applications
- Observative Study

ABSTRACT

Introduction

Electronic patient-reported outcomes (ePROs) have tremendous potential to optimise palliative and supportive care for children with cancer, their families and healthcare providers. Particularly these children and their families are subjected to multiple strains caused by the disease and its treatment. The MyPal digital health platform is designed to address these complex demands by offering pursuant ePRO-based functionalities via two mobile applications, one developed for children and the other for their parents.

Methods and Analysis

In this observational prospective feasibility study, 100 paediatric oncology patients aged between 6 and 17 years and at least one of their parents/legal guardians will be recruited at three clinical sites in two European countries (Germany and Czech Republic). They will use the mobile applications which are part of the novel digital health platform. During a 6-month study period, participants will complete various ePROs via the applications addressing quality of life, satisfaction with care and impact of the disease on the family at monthly intervals. Additionally, priority-based symptom reporting is integrated into a serious game for children. Outcomes that will be assessed concern the feasibility and the evaluation of the newly designed digital health platform to contribute to the evidence base of clinical ePRO use in paediatric oncology and palliative care process.

Ethics and Dissemination

The MyPal-Child study obtained ethical approval from the Ethics Committee responsible for the University of Saarland, i.e. the Ärztekammer des Saarlandes, the Ethics Committee of the Medical School Hannover and the Ethics Committee of the University of Brno. Study results will be disseminated through scientific publications, presentations at international conferences, congresses and a final report to the European Commission. General publicly accessible information can be found on the project website (www.mypal-project.eu) and social media.

For peer review only

Article Summary

Trial Registration Number

Universal Trial Number (UTN): U1111-1251-0043

German Clinical Trials Register (DRKS): DRKS00021458

ClinicalTrials.gov: NCT04381221

Strengths and Limitations of this study

- Multicentre (3 clinical sites in 2 European countries) observational study on the feasibility and acceptability of using ePRO-based systems in palliative care for paediatric oncology patients.
- Patient outcomes are reported independently in an inpatient/outpatient setting and the impact of this approach will be evaluated by the treating healthcare professionals.
- The design of the study is the product of a joint effort of interdisciplinary expertise from clinicians, palliative care experts, software engineers and scientists.
- The regular review of study participants' reportings demands healthcare professionals' additional time. The investigation of this factor is considered as a secondary objective in the elucidated study.
- Future randomised controlled trials are required to investigate the effectiveness of ePROs in paediatric palliative care management and treatment.

1. Introduction

According to the WHO definition, paediatric palliative care for children and their families is defined as "the active total care of the child's body, mind and spirit, and also involves giving support to the family. It begins when illness is diagnosed, and continues regardless of whether or not a child receives treatment directed at the disease. Health providers must evaluate and alleviate a child's physical, psychological, and social distress. Effective palliative care requires a broad multidisciplinary approach that includes the family and makes use of available community resources; it can be successfully implemented even if resources are limited. It can be provided in tertiary care facilities, in community health centres and even in children's homes" [1]. Unsurprisingly, this definition applies to children with cancer who require complex multimodal anticancer treatments but also benefit from palliative care supporting both the patients and their families to manage symptoms of the disease, side effects of treatment as well as treatment-related toxicity. Palliative care is not limited to end-of-life treatment

For peer review only

but preferably starts close to the time of diagnosis and continues during as well as outside the acute disease treatment phase to improve the quality of life (QoL). Such an early integration of palliative care either along with anticancer treatment or even as the only means of treatment in rare cases, can improve both patient and caregiver outcomes [2]. The latter is particularly relevant for parents and siblings of children or adolescents with cancer who are also impacted by the disease, its treatment as well as its prognostic uncertainty.

As children with cancer and their carers often believe that the adverse effects of anticancer treatment are inevitable, they often downplay symptom severity during clinical interviews [3], it is therefore possible, that more accurate assessments can optimise symptom reporting and generate richer and more reliable data. In that respect, major improvements can be anticipated through standardising patient-reported outcomes (PROs), defined as "measurements based on a report that comes directly from the patient about the status of a patient's health condition without amendment or interpretation of the patient's response by a physician or anyone else" [4, 5].

Overall, the use of PROs has become a prominent topic in healthcare innovation, highlighting the role of the patients' experience of their status as a key measure of healthcare quality, especially in cancer [6]. PROs provide a more comprehensive view on the patient's experience outside the environment of care and complement data gathered by HCPs or physicians. On these grounds, solutions are evidently required for facilitating the use of PROs in routine clinical practice of paediatric oncology and palliative care [7, 8], and these can be offered, amongst others, by information and communication technologies (ICT). Published evidence also supports the incorporation of electronic PRO (ePRO) assessments into healthcare as these have the potential to improve the quality of care delivered to patients with cancer [9].

MyPal is a Horizon 2020 Research & Innovation Action aiming to foster palliative care for people with cancer by leveraging ePRO systems through their adaptation to the personal needs of the person with cancer and his/her caregiver(s). It aspires to empower people with cancer and their carers in capturing more accurately their symptoms/conditions, communicate them in a seamless and effective way to their healthcare providers and, ultimately, foster action through advanced methods of identification of important deviations relevant to the patient's state and QoL. Providing this information in a timely and comprehensive manner throughout the disease course will reinforce the potential of applying a patient-centred and integrated palliative care approach for cancer patients with the participation of all relevant healthcare professionals (i.e. oncologists, specialised physicians, psychologists, nurses) to cope with the specific disease.

In order to accomplish its mission, MyPal will exploit technological advances in ICT for supporting patients, family members and healthcare providers through a systematic and comprehensive ePRO-

For peer review only

based digital health platform. It will demonstrate and validate the proposed add-ons to care for two different patient groups, i.e. adults suffering from haematological malignancies and children with solid tumours or leukaemia, hence targeting different age groups and cancer types, through two carefully designed clinical studies that will be conducted in diverse healthcare settings across Europe. The observational study with children with cancer (namely MyPal4Kids) is the subject of the presented protocol.

2. Methods and Analysis

Study Design

This is a multi-national non-experimental observational prospective feasibility study, enrolling paediatric oncology patients as well as at least one of their parents or legal guardians at 3 clinical sites in Europe. The emphasis is on data collection from the study participants during their 6 months of study enrolment via the functionalities of the newly developed MyPal digital health platform. The methodological approach involves two main mobile applications (see Section MyPal Digital Health Platform). The first comprises of a so-called 'serious game' developed with the aim to combine the advantages of modern ePROs with the motivational aspects of gaming by wrapping symptom reporting in the entertaining cover of an age-appropriate, non-violent game. Such an approach of gamification follows recommendations to encourage engagement of young patients and to split up otherwise long assessment questionnaires [10].

The second concerns convenient self- and proxy reporting for carers, i.e. in the context of this study the child's parents/legal guardians and healthcare providers, via ePROs as outcome measures. The study aims to evaluate the feasibility of integrating the developed ePRO-based system for palliative care in children with cancer into paediatric oncology care and assessing its benefit and support for the child and their parents. Study participant recruitment has started in December 2020 with an estimated study completion in March 2022.

Objectives and Outcome Measures

Primary Objective

The main objective is to assess the feasibility and acceptability of a comprehensive, patient-centred service for palliative care in peadiatric oncology by adapting and advancing ePRO systems.

The corresponding primary outcome measures are the rates of recruitment, participation, adherence and premature discontinuation to the different components of the MyPal digital health platform

For peer review only - http://bmjopen.bmj.com/site/about/guidelines.xhtml

during study enrolment. Further primary outcome measures are quantitative and qualitative data to be collected as follow-up to study enrolment: quantitative data from parents through the standardised System Usability Scale (SUS) [11] as well as from the children through a newly adapted version of the SUS; qualitative data through structured interviews and focus groups with participants to identify further barriers, facilitators, preferences and engagement with regards to the MyPal digital health platform as well as differences between the participating clinical sites.

Secondary Objectives

Secondary objectives of this study are to demonstrate the appropriateness and acceptability of several ePRO assessment tools, specifically:

- of children's symptom burden through a novel digital adaptation of the validated print versions of the Mini-SSPedi and SSPedi questionnaires [12, 13] as secondary outcome measure during study enrolment.
- of children's QoL under cancer treatment through the PedsQL™ Cancer Module [14, 15] as a secondary outcome measure during study enrolment.
- of parents' burden through the Impact on Family Scale as a secondary outcome measure during study enrolment, specifically with regards to: financial impact, family-social impact and personal strain for the primary family carer [16].
- of the QoL of parents having a child with cancer to be assessed through the EQ-5D-3L [17] as a secondary outcome measure during study enrolment. The parents' QoL will be evaluated with regards to the following dimensions: mobility, self-care, usual activities, pain/discomfort and anxiety/depression.
- of parents' satisfaction with the care offered to their children with cancer using the EORTC PATSAT C33 [18, 19] as a secondary outcome measure during study enrolment, which assesses patients' perception of the quality of medical and nursing care, as well as the organisation of care and services of an oncology department. It has been adapted appropriately to assess parents' satisfaction with children's cancer care in agreement with the authors of the questionnaire.

Further secondary objectives are:

- to determine the usage and evaluation of the MyPal apps including the gamified ePRO by children with cancer. This objective is linked to both the primary outcome measures as well as to EORTC PATSAT C33 as secondary outcome measure.

For peer review only

- to determine the impact and effect on healthcare professionals in two European countries due to the integration of ePROs in palliative care. The corresponding secondary outcome measure is a specifically developed web-based online questionnaire to be completed as follow-up to study enrolment, evaluating strain parameters such as additional time spent on care, usability, user-experience etc.
- to contribute to the evidence-base of the effectiveness of ePROs in palliative care and paediatric oncology which is linked to Mini-SSPedi/SSPedi and to the developed web-based online questionnaire as secondary outcome measures.

Patient Recruitment

In total, 100 children with leukaemia or solid tumours and their parents will be prospectively enrolled in the study across all 3 participating clinical sites:

1. University of Saarland (Germany)
2. Hannover Medical School (Germany)
3. University Hospital Brno (Czech Republic)

At each clinical study site, participants will be screened for eligibility and contacted by the local study team (see Box 1 for eligibility criteria). Information sheets will be handed to the parents and their child (see supplementary appendix 1). For each eligible participant, informed consent will be sought prior to any study-related activities (see supplementary appendix 2). As far as paediatric patients are concerned, information sheets and consent forms have been designed according to different age groups included in the study. Parents and children will be given an appropriate time of at least 24 hours to consider participation and to ask follow-up questions.

BOX 1 Eligibility Criteria

Inclusion Criteria for Children

- 6-17 years of age
- Diagnosed with paediatric leukaemia or solid cancer in the past 12 months
- Receiving anti-cancer treatment at one of the participating clinical sites
- Have age-appropriate speaking, reading and comprehension skills in either the German or the Czech language
- Access to an internet connection and mobile device (e.g. smartphone or tablet)

Inclusion Criteria for Parents

For peer review only

- Parent(s) with a child eligible for the study, as per the inclusion and exclusion-criteria
- Ability to speak, read and understand German or Czech language
- Access to an internet connection and mobile device (e.g. smartphone or tablet)

Exclusion Criteria for Children and Parents

- Anyone who is not able to participate in the study according to the clinical judgment of the site chief investigator or any other authorised person of the research team. This judgment has to be documented for each child/parent not being enrolled.

For peer review only

The patients' and parents' written consent constitutes the start of study enrolment. During the 6 months of study enrolment, children will be asked to complete questionnaires implemented as ePROs and integrated into the serious game via the MyPal platform both at baseline and at regular time intervals after receiving an introductory session by a research team member and in-app tutorials (see Figure 1). Parents as well as HCPs can help the children and complete the questionnaires as proxies throughout the study. Parents will also complete periodic questionnaires at baseline and subsequently using an app provided by the MyPal digital health platform. Independently, the medical care of the child will continue as required throughout and after the course of the study, e.g. anticancer treatment, receiving palliative and/or supportive care. After 6 months of study enrolment, children will be offered to continue playing the game without further data reporting or questionnaires via the MyPal digital health platform. Likewise, data will no longer be collected from their parents. Seven days after the end of data reporting, paper-based follow-up surveys will be completed by the children and their parents to evaluate their judgment on the MyPal platform and its usability.

HCPs will be asked to evaluate the impact and usability of the MyPal platform by a web-based online questionnaire to be completed at the end of the study or earlier if circumstances, e.g. leaving a job, suggest it.

MyPal Digital Health Platform

The MyPal digital health platform and the usage of its applications is the central innovation introduced and tested for feasibility and acceptability in this study. A participatory design was adopted in the development of the digital health platform involving a series of focus groups and discussions with patients and parents in order to identify needs and preferences as well as to validate tools, assess user

experience etc. The usage of the MyPal platform revolves around the reporting of physical symptoms caused by the disease and medication as well as QoL of the child with cancer via the MyPal Child app. Secondarily it comprises the reporting of their parents or legal guardians via the MyPal Carer app. The types of users of the MyPal digital health platform are specified in Table 1 while the software and hardware modules of the system are presented in Figure 2.

Table 1: The primary and secondary users of the MyPal digital health platform

User type	Description
Children with cancer	The child cancer patients registered at the participating clinical centres (primary users)
Parents of children with cancer	The parents or legal guardians of the primary users (secondary users)
Healthcare professionals (HCPs)	The interdisciplinary team of treating clinicians (oncologists, haematologists) nurses, psychologists, social workers, other palliative care members) of participating clinical centres (secondary users)

Other parameters (such as satisfaction with care, impact of disease on family, parents' QoL) are also reported by the parents via the MyPal Carer app. The symptom-related information is delivered instantly to the account of the treating HCP and can be visualised within a MyPal web-based application. The usage of the MyPal functionalities is described in more detail below from the standpoint of the patient, the parent and the HCPs.

Children's involvement

The game is designed for a target group from 6 to 17 years old, requiring basic reading skills. Voice-over is offered to support younger children. The art style is defined by a colourful underwater exploration (see Figure 3), and specifically designed to appeal to a wide age range. Designed as a runner game, the game character is continuously running through levels of a game environment with collectable items and obstacles while being controlled by the player. Runs are paused to answer single symptom-related questions inside the game (see Table 2). Factors for long- and short-term motivation, e.g. collectibles and rewards, high scores and customisable game character have been incorporated into the game to make the regular use of the app and the symptom assessment as an integrated ePRO appealing and entertaining over a long period of time.

For peer review only

Registration Phase. Registration requires actions both in the MyPal Child app and the MyPal web-based app by the HCP. During this phase (1) the patient is registered into the MyPal digital health platform by an HCP or research team member, e.g. research nurse, who also helps with the initial login; (2) a number of preferences are set; (3) baseline assessment of the patient's physical symptoms are collected. In the MyPal Child app, the patient receives a short training session and an in-app tutorial helps to get familiar with the functionalities.

Main usage phase. The main usage phase lasts for 6 months, during which the patient is given access to all the functionalities of the MyPal Child app, outlined in Table 2. The first three functionalities can be accessed by the patient at any point in time, while the last is activated/deactivated automatically within the MyPal digital health platform on a monthly basis.

Table 2: The functionalities of the MyPal Child app offered to the child patients

	Functionality	Description
**P1**	Game playing	This is a prominent functionality of the MyPal gamified app. The patient is offered to play 3 new run sessions in a day, during which he/she controls a diver swimming in an underwater world. The patient can also customise various elements of the game (e.g., the appearance of the diver) at any time.
**P2**	In-game symptom reporting	This is the main ePRO data collection functionality of the MyPal gamified tablet app. During each gaming session, the game pauses 5 times for the user to answer a single symptom-related question (see Figure 3); each question is seamlessly integrated in the flow of the game (the patient still sees the diver in his/her underwater environment). The set of questions to be asked stem from the unique digital adaption based on the validated SSPedi/Mini SSPedi paper-version questionnaire. Each question to be asked during a game session is determined by an internally validated novel question prioritisation algorithm.
**P3**	Spontaneous symptom reporting	This is the secondary functionality that allows patients to report physical symptoms whenever they wish. An electronic implementation of the SSPedi/Mini SSPedi questionnaire outside the game is employed for the spontaneous reporting.

For peer review only - http://bmjopen.bmj.com/site/about/guidelines.xhtml

**P4**	Periodic QoL reporting	This is the secondary functionality that asks patients to report their QoL once per month. An electronic implementation of the validated PedsQL questionnaire is therefore employed.

Parent's involvement

The parents of children with cancer interact with the MyPal reporting smartphone app in 2 sequential phases, which are outlined below.

Registration Phase. During this phase (1) the parent is linked to their child within the MyPal digital health platform; (2) baseline assessment of a subset of the outcome measures of the study via self-reporting are collected. Parents undergo a short training session and are guided during registration by an HCP participating in the study, e.g. research nurse.

Main usage phase. The main usage phase lasts for 6 months and is aligned with the patient participation in the study. The only functionality that is offered to the parent is reporting, either as a proxy or for themselves, concerning a subset of the outcome measures of the study. The reporting functionality of the MyPal Carer app is outlined in Table 3.

Table 3: The functionalities of the MyPal Carer app offered to the child's parents

	Functionality	Description
**R1**	Patient QoL reporting	Proxy reporting of the QoL for the patient using an electronic implementation of the validated PedsQL questionnaire. Older patients will be asked to complete the questionnaire directly in the MyPal Child app. This is linked with an outcome measure of the study and is reported once per month.
**R2**	Parent QoL reporting	Reporting of the QoL of the parent using an electronic implementation of the validated EQ-5D-3L questionnaire. This is linked with an outcome measure of the study and is reported once per month.
**R3**	Satisfaction with care reporting	Reporting of the satisfaction with received care using an electronic implementation of the validated PATSAT-C33 questionnaire. This is linked with an outcome measure of the study and is reported once per month.

For peer review only - http://bmjopen.bmj.com/site/about/guidelines.xhtml

**R4**	Disease impact on family scale reporting	Reporting of the impact of the disease on the family using an electronic implementation of the validated Impact on Family Scale questionnaire. This is linked with an outcome measure of the study and is reported once per month.
**R5**	Patient Symptom Reporting	Proxy reporting of symptoms for the patient using an electronic adaption of the SSPedi/Mini-SSPedi questionnaire. Usually patients will be asked to answer questions with regard to symptoms directly in the gamified app.

HCP's involvement

Registration Phase. After a short registration procedure that takes place during their first visit to the web-based app, the HCPs can continuously interact with it throughout the duration of the study. HCPs are given a short in-app training session.

Main usage phase. During the main usage of the MyPal web-based app, the HCPs can access symptom related data being stored in the system backend which had been collected by (1) the MyPal Child app and (2) the MyPal Carer app. HCPs are authorised to solely access the data of the patients of their associated clinical centre. The individual data of participating patients are reviewed by the associated HCP at least once every 72 hours to check for new entries. The data review and any action related to this are recorded through the web interface. The functionalities of the MyPal web-based app are outlined in Table 4. All of them can be accessed by the HCP at any time. In addition, the HCP can use the MyPal Carer app in order to complete questionnaires as a proxy for one of their patients.

Table 4: The functionalities of the MyPal web-based app offered to the HCPs

	Functionality	Description
**H1**	Incoming information summary	A central page of the web-based app displays in a summarised form the incoming patient information that has not been reviewed yet. Incoming information is automatically prioritised in the system using custom algorithms whereby pieces of incoming information are assigned the highest priority and placed at the top of the list. Whenever an item is reviewed in full, it is removed from the list.
**H2**	Individual data dashboard	A page that presents all the information collected for a given patient since the start of their participation in the study, using a dashboard with modern

For peer review only

		visualisations (see Figure 4). The information includes primarily the patient's responses to the symptom questionnaire along with additional clinical information (e.g., treatment, age group, diagnosis group etc.).
**H3**	Symptom Status over Time	This functionality and its visualisation are linked to the individual data dashboard. HCPs can document interim reports on current or past symptoms at different points in time as well as corresponding measures taken via selection of pre-defined choices. Additionally, the current or retrospective status of the patient's disease as well as the goal of treatment can be updated considering the whole course of the study.
**H4**	Aggregated data dashboard	A page that presents aggregated and summarised information coming from all patients that participate in the study (descriptive statistics such a min, max, average and percentiles) using an analytics dashboard with modern visualisations.
**H5**	HCP response log	A page that is used for logging potential responses (e.g., referral to a specialist or prescription of medical examinations) of the HCP to the presented information of a specific patient. The HCP can log any actions taken after visiting individual data dashboard of a patient in a structured manner.

Data Collection and Analysis

All applicable national and EU legislation, particularly the General Data Protection Regulation (EU) 2016/679 [20] for the protection of individuals have been considered in the design of the study-related IT infrastructure with regard to the confidential processing, collection and access to personal data. The technical deployment of the MyPal platform comprises of local server installations at each of the clinical study sites and one central server installation at the sponsor's site distinctly defining premises of data accessibility. The data security concept entails regular synchronisation of only anonymised data from local to central database. The apps being installed on mobile devices are protected by confidential credentials and store encrypted data only temporarily locally in case of absent internet connection until they are deleted from the mobile device subsequent to transmission to the respective databases.

For peer review only

Sample size calculation

Assuming relatively acceptable values for the attrition rate (i.e., 20%) and the missing data (i.e., 30%), the sample size analysis concluded that 100 recruited paediatric patients providing one measure at enrolment (baseline) and 6 repeated measures (at Months 1, 2, 3, 4, 5, 6) are sufficient for the power of the intended statistical testing to be over 90% in all cases, given (a) a 0.05 significance level, and (b) an effect size of 0.1; the employed value of the effect size was based on a priori knowledge of the domain, all power calculations were performed using the G*Power statistical analysis software [21].

Data Analysis

The subsequent analysis and evaluation will serve to assess the feasibility of the MyPal digital healthcare platform which uses ePROs to be ideally beneficial and supportive for the child and parents in palliative care.

Subgroup analysis of the outcome measures will be performed at baseline, Month 3 and Month 6 of the study using one-, two- and three-way ANOVA in order to detect potential differences between specific groups of participants. The level of significance for all statistical tests is set to a=0.05, in accordance with the power calculations. The grouping variables that will be assessed are (a) the clinical centre (origin), (b) the country of residence, (c) the age group, (d) the disease category (leukaemia, solid tumour, brain tumour), (e) the disease stage and (f) the starting point of the study in relation to the date of diagnosis. The latter point refers to recruited patients who have been under treatment for more than six months receiving care at the respective participating clinical site. These patients constitute a cohort for retrospective baseline data collection: the data collected from their completion of the ePROs at the beginning of their study enrolment will later be compared to outcome measure data derived from prospectively recruited patients whose treatment started within the last 6 months. Both groups will be followed likewise during the course of the study. Further data, like the daily number of steps (estimated by the smartphone/tablet using the measurements of the built-in sensors) as well as the documentation of the symptom status over time, will be used to examine the feasibility to assess for correlations with reported symptoms and reported physical activity.

To evaluate the changes in outcome measures over time in the child cohort, repeated measures analysis of variance (ANOVA) will be applied on each measure (or a non-parametric equivalent). Post-hoc analysis will be applied as appropriate. Since wording and layout of questionnaires were partly modified compared to the validated versions for electronic completion and gamification, another focus

For peer review only

is (or would be) to assess the feasibility of such modifications to the original questionnaires. Repeated measures multivariate ANOVA (MANOVA) will be applied to the QoL of the children and the parents which serve as a pair of dependent variables.

To examine potential QoL differences between the participating sites, independent-samples t tests and analysis of covariance (ANCOVA) models will be applied that will control for baseline criterion scores and potential confounders such as age group and sex which may be imbalanced between groups and associated with outcomes of interest.

The potential effect of the attrition is going to be assessed according to the methods introduced in Fewtrell *et al*. [22], e.g., comparison of baseline characteristics of seen versus not seen at the follow-up participants to assess potential bias, sensitivity analysis, re-evaluation of the power of the study with the attained sample size. The impact of missing data in the attempted analysis is going to be evaluated based on the importance of the variables. Checks for data “missing at random” and “missing completely at random” are going to be performed.

Additional quantitative feedback will be gathered by a final evaluation of the MyPal digital health platform based on an appropriate usability one-time scale, i.e. system usability scale for patients or parents, whereas HCPs’ feedback will be collected via a designed web-based questionnaire at the end of the study.

3. Ethics and Dissemination

The integration of ePROs dealing with health issues into mobile applications raises ethical aspects which have been a crucial pillar in the implementation plan of this project under the monitoring of an internal ethics committee. Particular attention has been paid regarding data protection and the design of the MyPal-Child study protocol as it involves children being a vulnerable user group. The study protocol has obtained ethical approval from the ethics committee competent for the University of Saarland: Ärztekammer des Saarlandes (reference: Ha 23/20), the ethics committee of the Medical School Hannover (reference: 9095_BO_K_2020) as well as the ethics committee of the University of Brno (reference: 01-120220/EK). The study results will be disseminated after study completion through publications in scientific journals, presentations at scientific conferences and congresses as well as a final report to the European Commission. Information on the MyPal project and newsletters, are publicly accessible via the project website (www.mypal-project.eu), Twitter (@H2020MyPal) and Facebook (@MyPalProject). Based upon study findings, concepts and strategies for the continuation of the MyPal platform with its tools and game as well as follow-up studies are considered.

For peer review only

Concluding Remarks

The foreseen advancement of the presented MyPal's patient-centred ePRO approach will offer a significant opportunity for children with cancer, their parents and healthcare providers to actively participate in the care process. By exploiting technological advances in information and communication technologies, MyPal aspires to contribute to bridge the gap between timely reporting and tracking of symptoms and the personalised actions performed by healthcare providers addressing the patient's needs which can vary across the disease course. The paradigm shift from passive patient reporting to active patient engagement could both enhance palliative and supportive care for children with cancer and improve coping with the disease.

Author Contributions

MM, NG, CK, CM, AS, SP, LS, PL drafted the study protocol
MM and CK contributed equally as main authors on the manuscript
NG, TG, SP, AS, JD, CM, KS, JL, LS, CP contributed to critical revisions of the study protocol and the manuscript.
NG, AS and PL are the Site Chief Investigators and take overall responsibility for all aspects of the study design, the protocol and the study conduct at the involved clinical study sites.

Acknowledgments

MyPal is coordinated by Dr Kostas Stamatopoulos of the Centre for Research and Technology Hellas, Greece. Other partners are: Fraunhofer Institute for Biomedical Engineering, Germany; Foundation for Research and Technology Hellas, Greece; International Observatory on End of Life Care, Lancaster University, UK; Central European Institute of Technology, Masaryk University, Czech Republic; Karolinska Institute, Sweden; Vita-Salute San Raffaele University, Italy; University Hospital of Heraklion, Greece; Hannover Medical School, Germany; University Hospital Brno, Czech Republic; Saarland University, Germany; Promotion Software GMBH, Germany; Atlantis Healthcare, UK; European Association for Palliative Care; International Children's Palliative Care Network; National School of Public Health, Greece. The authors are grateful to the clinicians at the clinical study sites who are essential to this study (patient recruitment, symptom assessment and management and data collection). This work is dedicated to Vassilis Koutkias.

Funding

MyPal: Fostering Palliative Care of Adults and Children with Cancer through Advanced Patient Reported Outcome Systems, is funded by the Horizon 2020 Framework Programme of the European Union under Grant Agreement Nr. 825872.

For peer review only

Competing interests

None declared.

Provenance and peer review

Not commissioned; externally reviewed.

Figure Legends

Figure 1: Patient-specific study enrolment schema of MyPal4Kids.

Figure 2: Software and hardware modules of the MyPal digital health platform. The MyPal Child and Carer app are useable via various types of mobile devices. The user of the MyPal Carer app are the parents/legal guardians involved in the study. Healthcare professionals can use the MyPal Carer app as well to report as proxy for the child.

Figure 3: A) A screenshot of the gamified symptom questionnaire as part of the MyPal Child app. B) Screenshots taken of the MyPal Carer app depicting the PATSAT C-33 questionnaire with instruction screen, selection of patient setting screen and one sample question screen.

Figure 4: Symptoms reported by study participants can be visualised in the MyPal web-based app for HCPs to be reviewed according to adjustable settings.

For peer review only

References

[1] World Health Organization. (1998). Cancer pain relief and palliative care in children. World Health Organization

[2] Hoerger M, et al. Defining the Elements of Early Palliative Care That Are Associated With Patient-Reported Outcomes and the Delivery of End-of-Life Care. J Clin Oncol. 2018;36(11):1096-102.

[3] Leahy AB, Feudtner C, Basch E. Symptom monitoring in pediatric oncology using patient-reported outcomes: why, how, and where next. The Patient-Patient-Centered Outcomes Research. 2018 Apr 1;11(2):147-53.

[4] US Food and Drug Administration. Guidance for industry: patient-reported outcome measures: use in medical product development to support labelling claims: draft guidance. Health Qual Life Outcomes. 2006; 4:79.

[5] Eliasen A, Abildtoft MK, Krogh NS, Rechnitzer C, Brok JS, Mathiasen R, Schmiegelow K, Dalhoff KP Smartphone App to Self-Monitor Nausea During Pediatric Chemotherapy Treatment: User-Centered Design Process. JMIR Mhealth Uhealth 2020;8(7):e18564

[6] LeBlanc TW, Abernethy AP. Patient-reported outcomes in cancer care - hearing the patient voice at greater volume. Nat Rev Clin Oncol. 2017;14:763-72.

[7] Downing J, Namisango E, Harding R. (2018) Outcome measurement in paediatric palliative care: lessons from the past and future developments. Annals of Palliative Medicine. doi: 10.21037/apm.2018.04.02

[8] Bausewein C, Daveson BA, Currow DC, Downing J, Delians L, Radbruch L, Defilippi K, Lopes Ferreira P, Costanini M, Harding R and Higginso IJ. (2015) EAPC White Paper on outcome measurement in palliative care: Improving practice, attaining outcomes and delivering quality services – Recommendations from the European Association for Palliative Care (EAPC) Task Force on Outcome Measurement. Palliative Medicine.

[9] Maguire R, et al. The eSMART study protocol: a randomised controlled trial to evaluate electronic symptom management using the advanced symptom management system (ASyMS) remote technology for patients with cancer.BMJ Open. 2017;7(5):e015016.

[10] Peres SC, Pham T, Phillips R. Validation of the System Usability Scale (SUS). In: Proceedings of the Human Factors and Ergonomics Society Annual Meeting, 2013; 57(1):192–196.

[11] Jupp, JC, Sultani, H, Cooper, CA, Peterson, KA, Truong, TH. Evaluation of mobile phone applications to support medication adherence and symptom management in oncology patients. Pediatr Blood Cancer. 2018; 65:e27278. https://doi.org/10.1002/pbc.27278

[12] Tomlinson, Deborah; Hyslop, Shannon; Stein, Eliana; Spiegler, Brenda; Vettese, Emily; Kuczynski, Susan et al. Development of mini-SSPedi for children 4-7 years of age receiving cancer treatments. In: BMC cancer; 2019; 19(1):32.

[13] Dupuis LL, Johnston DL, Baggott C, Hyslop S, Tomlinson, D, Gibson P, et al. Validation of the Symptom Screening in Pediatrics Tool in Children Receiving Cancer Treatments. In: Journal of the National Cancer Institute 2018; 110(6): 661–668.

[14] Varni JW, Burwinkle TM, Katz ER, Meeske K, & Dickinson P. The PedsQL in pediatric cancer: Reliability and validity of the Pediatric Quality of Life Inventory Generic Core Scales, Multidimensional Fatigue Scale, and Cancer Module. Cancer, 2002; 94:2090-2106

[15] Robert RS, Paxton RJ, Palla SL, Yang G, Askins MA, Joy SE, Ater JL. Feasibility, reliability, and validity of the pediatric quality of life inventory™ generic core scales, cancer module, and multidimensional fatigue scale in long-term adult survivors of pediatric cancer. Pediatric Blood & Cancer 2012; 59:703–707.

[16] Stein REK & Riessman CK. The development of the Impact on Family Scale: Preliminary findings. Medical Care, 1980; 18.465-472.

[17] EuroQol Research Foundation. EQ-5D-3L User Guide, 2018. Available from: https://euroqol.org/publications/user-guides. Last accessed: October 1, 2019.

For peer review only

[18] Bredart A et al. “Phase III study of the European Organisation for Research and Treatment of Cancer satisfaction with cancer care core questionnaire (EORTC PATSAT-C33) and specific complementary outpatient module (EORTC OUT-PATSAT7).” European Journal of Cancer Care (English Language Edition); 2018; 27(1).

[19] Bredart A et al. “The European organization for research and treatment of cancer – satisfaction with cancer care questionnaire: revision and extended application development.” Psycho-Oncology 2017; 26(3): 400-404.

[20] Regulation (EU) 2016/679 of the European Parliament and of the Council of 27 April 2016 on the protection of natural persons with regard to the processing of personal data and on the free movement of such data, and repealing Directive 95/46/EC (General Data Protection Regulation) (Text with EEA relevance) Official Journal L119, 4.5.2016:1–88.

[21] Faul F, Erdfelder E, Lang AG, & Buchner A. G*Power 3: A flexible statistical power analysis program for the social, behavioral, and biomedical sciences. Behavior Research Methods, 39. 2007;175-191

[22] Fewtrell MS, Kennedy K, Singhal A, Martin RM, Ness A, Hadders-Algra M, Koletzko B, Lucas A. How much loss to follow-up is acceptable in long-term randomised trials and prospective studies?. Archives of Disease in Childhood. 2008;93(6):458-61.

For peer review only

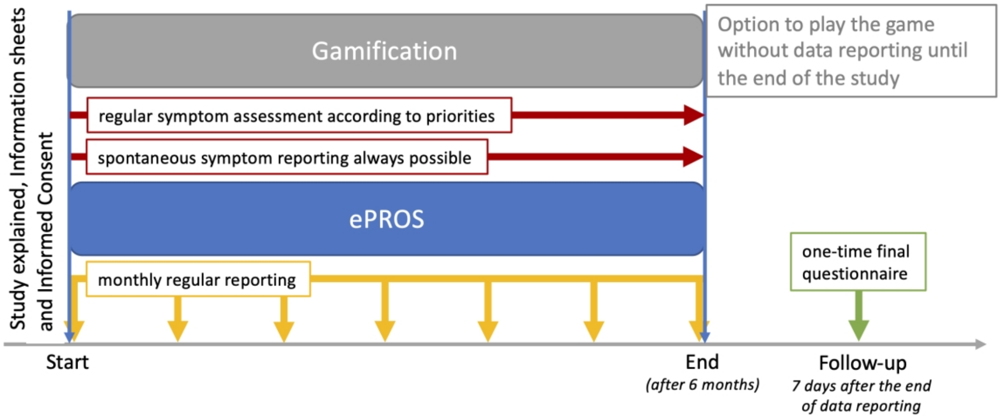

Patient-specific study enrolment schema of MyPal4Kids.

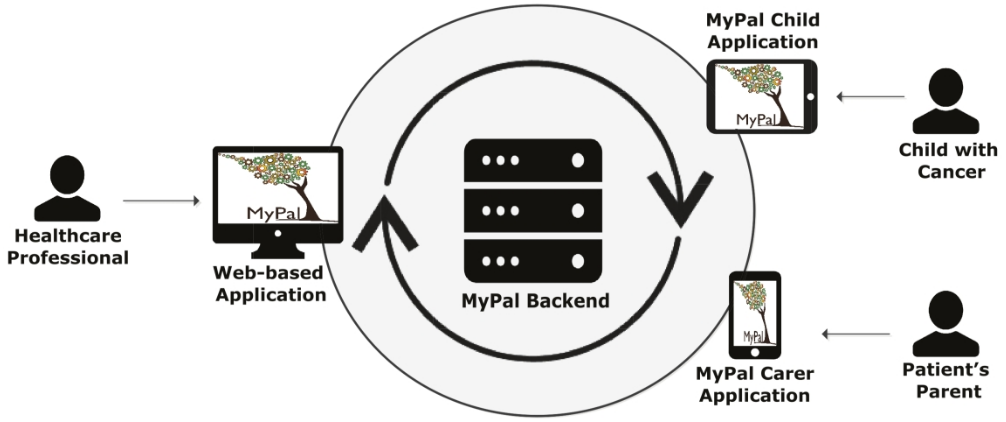

Software and hardware modules of the MyPal digital health platform. The MyPal Child and Carer app are useable via various types of mobile devices. The user of the MyPal Carer app are the parents/legal guardians involved in the study. Healthcare professionals can use the MyPal Carer app as well to report as proxy for the child.

239x101mm (300 x 300 DPI)

A 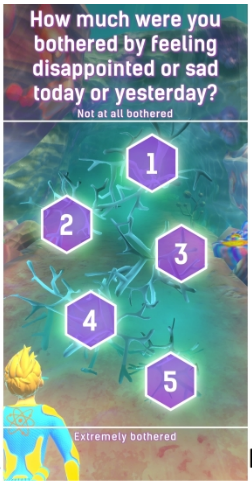

B 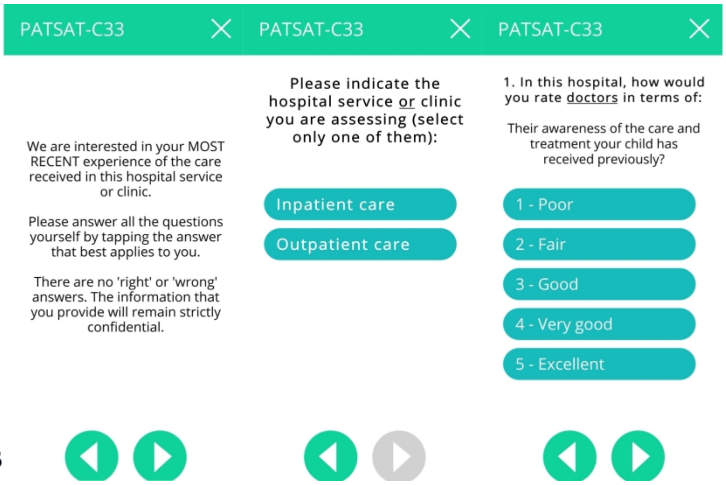

A) A screenshot of the gamified symptom questionnaire as part of the MyPal Child app. B) Screenshots taken of the MyPal Carer app depicting the PATSAT C-33 questionnaire with instruction screen, selection of patient setting screen and one sample question screen.

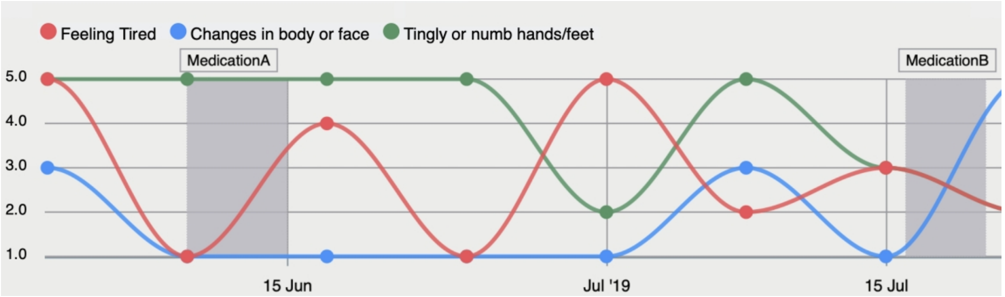

Symptoms reported by study participants can be visualised in the MyPal web-based app for HCPs to be reviewed according to adjustable settings.

Informed Consent Form for Parents / Legal Guardian

- I consent to use Internet connected devices, e.g. smart phone or tablet, to complete self-reporting questionnaires monthly via an app to assess well-being, satisfaction with cancer care received for my child and their impact on the family.
- I consent to respond one-time to a paper-based questionnaire 7 days after the 6 months of study enrollment to evaluate the usability of the MyPal platform.
- I consent that I have read and understand the information sheet explaining the above research project and I have had the opportunity to ask questions about it.
- I consent that information has been provided to me in a language that I fully understand.
- I am aware that NO biological samples will be collected from my child in the study and that the study does NOT entail the administration of any medication to my child.
- I am aware that the participation of my child is absolutely voluntary and that I am free to withdraw my consent at any time without giving any reason and without there being any negative consequences.
- I consent that if I withdraw myself or my child from the study after some data have been collected, these data will be anonymized but not deleted. No further data will be collected in this case.
- I am aware that in all research outputs (papers, presentations, articles, reports) the anonymity the data will be protected.
- I am aware that data collected during MyPal project will never be transferred to countries outside of the European Union.
- I consent that medical data that has been obtained during this project will be stored for 15 years, in (name of the clinical site) and the data that has been collected for the purposes of the study, will be kept in a de-identified form at the central installation of the sponsor of the study.
- I consent that health care professionals will report disease-related data of your child to the MyPal system.
- I am aware that any supportive information provided by health care professionals (e.g. physicians) via the MyPal platform does not imply legal liability and that MyPal is not designed as an emergency alert system and doctors might not respond immediately.
- I consent that I have understood the information sheet for the study including information on rights for affected person in terms of personal data. Hereby, I have obtained the contact details of the Data Protection Officer responsible, in case I have concerns or queries explicitly dealing with data protection or want to lodge a complaint due to this issue.

I have read and understood this consent form and I have been provided with information regarding the research study. I have been given a copy of the information sheet and of this consent form.

In case I have concerns of any kind or further questions, I may contact (enter contact details)

A copy of this agreement will be given to me and another copy will be retained for record keeping by the project.

○ **I agree for my child to participate in the study**

Name of the child: ______________________________________, Birthday: ___.___.20….

______________________	______________	______________________
Name of the Mother	Date	Signature
______________________	______________	______________________
Name of the Father	Date	Signature
______________________	______________	______________________
Name of the Researcher	Date	Signature

For peer review only - http://bmjopen.bmj.com/site/about/guidelines.xhtml

Version 1.1 – 05.03.2020

MyPal-Child Information Sheet for Parents / Legal Guardian

We would like to invite your child to participate in a study conducted by Dr (enter authorized clinician) and his/her team at the University of (enter Clinical Site). Before you decide to give permission for your child, we would like to help you understand the study and what is involved. Please take time to read the following information carefully and to decide whether or not you wish your child to take part.

In this study we are interested in exploring the use of digital technology that will help you (and possibly members of your family) to communicate your condition more accurately and effectively to your health care providers (i.e. oncologists, specialized physicians, psychologists, nurses). The aim is to improve the quality of care by using modern methods of individualized information, communication and support for patients with cancer the main focus of which is the patient (Patient-Reported Outcome). Supportive information provided by health care professionals via the application do not imply legal liability.

Aims of the study: The study in the context of the EU project MyPal[1] aims to propose a new system of care that is anticipated to improve quality of life in children with cancer and their parents, by exploring how digital technology can enhance communication, improve decision-making, emotional support and reinforcement of young patients and reduce symptom burden. It only involves observation of your child regarding the use of an application in a gamified form. It is important for you to know that the study:

- does not involve collection of biological samples
- does not involve administration of any medication
- does not aim to provide or change medical treatment solely based on the MyPal platform
- MyPal is not an emergency alert system and doctors may not respond immediately

Why has your child been chosen: Your child has been chosen to participate in this study because he /she is between 6-17 years of age and has been diagnosed with acute leukemia or solid cancer for which anti-cancer treatment is already being provided. Also, because he/she is fit to participate in the study and able to use an Internet connected device (smartphone or tablet). The contribution of children and adolescents is very important for the study to explore their specific needs and preferences and with the aim of making young people with cancer stronger to deal with their condition.

What does your child have to do: If you and your child agree to participate, your child will receive an introduction to get familiar with the MyPal App and its tools and how to use them. For the upcoming 6 months, your child can then perform the following tasks:

- Use the MyPal app through Internet connected devices, such as smart phone, tablet for a game which involves questionnaires about their well-being and symptoms. In addition, the physical activity of your child will be roughly estimated based on a daily step counter.
- With regard to the various questionnaires involved, the child will be free to choose not to answer any particular question or questions. Completion will be scheduled at baseline, and then monthly, some of them several times a week, until the end of the study. It is possible that these time intervals may change due to the needs of the study.
- Respond only once to paper-based questionnaire 7 days following the 6 months of study enrollment in order to evaluate the usability of the MyPal platform.

During the completion of the questionnaires your child may get assistance by you, a member of our research team or a health care professional.

Which tasks you as parents/legal guardian will be asked to complete:

- You will also receive an introduction on how to get familiar with and use the MyPal platform and its tools.
- Use the MyPal platform to complete monthly questionnaires about your well-being, satisfaction with your child's received care and the impact of the disease on you and your family.
- Help your child completing questionnaires if they ask you for help.
- respond only once to a paper-based questionnaire 7 days following the 6 months of study enrollment in order to evaluate the usability of the MyPal platform.

[1] Available from: https://mypal-project.eu/. Last accessed: January 20, 2019

For peer review only - http://bmjopen.bmj.com/site/about/guidelines.xhtml

Version 1.1 – 05.03.2020

Will the data collected in this project be kept confidential? Data protection is one of our most important priorities in this study. National laws on personal data protection will be implemented in order to guarantee the highest standards in personal data management. Further, all procedures for protecting personal information in this study are in accordance with the approved rules of the University of (enter clinical site) and with the European legislation including the General Data Protection Regulation 2016/679 (GDPR). Only data that is necessary for this research and no additional data will be collected. Our technology partners will provide technical support and tools such that data protection and security requirements are ensured. Any information, that is obtained in this study and that can be assigned to you or your child will remain confidential.

If you agree for your child to take part in this study, we will use his/her data in the ways needed to conduct our study and analyse the study results. Your rights to access, change or move your child's information are limited, as we need to manage this information in specific ways in order for the research to be reliable and accurate (cf. point d of Article 17(3) GDPR and Article 89 GDPR). If your child withdraws from the study, we will keep the information that we have already obtained and anonymize it for analysis at the end of the study. Anonymization means that the stored data is altered in such a way that it cannot be restored in its original form and all information which could link the collected data to you or your child is deleted. No more data will be collected after withdrawal.

We will not allow people from outside the project to know how you or your child answered the questions within the study. In case you have concerns or queries explicitly concerning data protection or you want to get into contact or lodge a complaint with data protection authorities, you can also contact the Data Protection Officer of our Research Institution: the corresponding contact details can be found on the last page.

Storage of data and security: Data will be stored in two ways, locally and centrally, which is elucidated in the following.

The app installed on the mobile device stores encrypted data, i.e. answers to questionnaires, only temporarily locally if the mobile device has no internet connection. As soon as an internet connection is available, the answers will be transmitted to the local database at your hospital and deleted from the mobile device. After the initial login to the app, an access token is stored on the device which facilitates each upcoming login.

The local database of (name of the clinical site) will store:

- responses to questionnaires about symptoms, spontaneous symptom reporting forms, etc.
- personal data (patient name, provenance, treating clinical site)
- clinical information (gender, age, diagnosis, disease stage/risk, treatment scheme, expected outcome, functional impairment, etc.). Concerning the clinical information, the (a) age and (b) diagnosis of the study subject are categorized before storage instead of precise detail.

The data included in the local database will be accessible only by the team of local investigators, and even the members of the team will only have access to the information needed for the specific user category with regard to the study. All personally identifiable data remain at any time at the (enter clinical site) and will neither be shared with the other participating clinical sites nor study sites. Furthermore, the data is protected against access by unauthorized people by username and password.

The central database which resides at the side of the sponsor of the study (Centre for Research and & Technology Hellas, Greece) and which fulfills all technical and organizational requirements for the safety and the security of the stored data to protect them against unauthorized people's access by access restriction. The central database will include a part of the local data (responses to the questionnaires and information needed to assess the endpoints of the study, e.g. quality of life, satisfaction with care etc.) which will be transferred from the participating clinical center to the central database identification to enable the joint analysis of data from the participating clinical sites. Before the transmission, personal data is removed (Anonymization). Anonymization is performed by removing the patient's name from the personal data and keeping among the clinical data only the gender, age group and categorized diagnosis information.

For peer review only - http://bmjopen.bmj.com/site/about/guidelines.xhtml

Version 1.1 – 05.03.2020

All data obtained in the study will be maintained for 15 years in the (enter name of the clinical site) where they have been collected or created.

Does your child have to participate? Participation is completely voluntary and you are free to consent or not to the participation of your child. Even if you agree, you remain free to withdraw this consent at any time without giving any reason and without this affecting your or the child's relationship with our research team or with the team providing clinical care. If you decide to withdraw from the study once data collection commenced, the collected data will not be erased, but will be retained in the study but in an anonymized form which will not in any way permit the child's or your identification. Once the research has been completed and the data been analysed, it will not be possible to withdraw this data from the study. No more data will be collected from you or your child after the withdrawal.

Are there possible disadvantages and/or risks in taking part? Participation of your child does not entail any risk of discomfort, pain, injury, illness or disease and we remind you that the study itself does NOT involve collection of biological samples or administration of any medication. However, if we come across an unexpected finding which we think requires notifying you, we plan to inform you, to discuss this in our team, and to consult with your child's treating physician. If you have any concerns please feel free to contact the Principal Investigator (see below for details) and once again, we would like to remind you that your participation is entirely voluntary. The health care professionals do not bear liability for information which are provided to the patients or parents via the Apps.

What are the possible benefits of taking part? You, your child, or your family may not receive any personal benefits from participating in the study except from the possibility to use and play the game. However, you may find participation in the study is a positive experience, through the use of the apps and by contributing to the improvement of the quality of life of young cancer patients in the process of their treatment.

Transfer of data: All data collected during the MyPal project will not be transferred to countries outside of the European Union.

What will happen to the results of the research project? The results will be used only for research purposes; they may be reported in research publications and may be made available to other researchers in an anonymized form, e.g. in presentations. In every research output (papers, presentations, articles, reports) the complete anonymity of your data will be ensured.

Right of the individual affected in terms of processing personal data

Based on the EU Data Protection Basic Regulation 2016/679 (GDPR), you are entitled the following data protection rights for individuals affected, which you can assert against the involved hospital operators.
You have the right of access to stored personal data relating to you (Article 15 GDPR). If you discover that incorrect data concerning your person is being processed, you can request correction or purpose-related supplementation (Article 16 GDPR).
You have the right to request the deletion of your data if there are specific reasons for deletion. This is particularly the case if they are no longer necessary for the purpose for which they were originally collected or processed (Article 17 GDPR).
You have the right to restrict the processing of your data, which means that your data will not be deleted but will be for data portability (Article 20 GDPR).
In principle, you also have a general right of objection to lawful data processing which is of public interest, in the exercise of official authority or on the basis of the legitimate interest of a site (Article 21 GDPR).
As already mentioned, however, these rights may be restricted in accordance with point d of Article 17(3) GDPR and Article 89 GDPR if they make it impossible or seriously impair the achievement of the study objectives.

Complaint to the supervisory authority in case of data protection violations

You have the right to forward a complain to the supervisory authority if you think that your personal data is being processed unlawfully. The address of the supervisory authority responsible for (specify clinical partner) is:

For peer review only - http://bmjopen.bmj.com/site/about/guidelines.xhtml

Version 1.1 – 05.03.2020

The State Commissioner for Data Protection (give details of the respective State Commissioner for Data Protection + insert address)

Contact with the data protection officer of (specify clinical partners) is as follows:

(please specify address, e-mail, telephone)

The contact to the local study director project at (indicate clinical partner) is as follows:
(indicate corresponding address/email/telephone number WITHOUT mobile phone number)

If you have any questions or concerns regarding the study, please do not hesitate to contact him/her at any time for further information.

Ethical approval: This study has obtained a positive vote by the Ethics Committee of the University of (enter clinical site) which means that the conduction of the study does not give rise to any objections from the ethical point of view.

For peer review only

For peer review only - http://bmjopen.bmj.com/site/about/guidelines.xhtml
